# Joint analysis of multiple high-dimensional data types using sparse matrix approximations of rank-1 with applications to ovarian and liver cancer

**DOI:** 10.1186/s13040-016-0103-7

**Published:** 2016-07-29

**Authors:** Gordon Okimoto, Ashkan Zeinalzadeh, Tom Wenska, Michael Loomis, James B. Nation, Tiphaine Fabre, Maarit Tiirikainen, Brenda Hernandez, Owen Chan, Linda Wong, Sandi Kwee

**Affiliations:** 1 University of Hawaii Cancer Center, 701 Ilalo Street, Honolulu, HI 96813 USA; 2 SNR Analytics, LLC, 45-115E Waikalua Road, Kaneohe, HI 96744 USA; 3 Department of Mathematics, University of Hawaii, Manoa, Honolulu, HI 96822 USA; 4 Interactive Biosoftware, Rouen, France; 5 The Hamamatsu/Queen’s PET (Positron Emission Tomography) Imaging Center, Queen’s Medical Center, Honolulu, HI 96816 USA

**Keywords:** Generalized singular value decomposition, Joint data analysis, Ovarian cancer, Hepatocellular carcinoma, The Cancer Genome Atlas, LASSO, Sparse signal detection

## Abstract

**Background:**

Technological advances enable the cost-effective acquisition of *Multi-Modal Data Sets* (MMDS) composed of measurements for multiple, high-dimensional data types obtained from a common set of bio-samples. The joint analysis of the data matrices associated with the different data types of a MMDS should provide a more focused view of the biology underlying complex diseases such as cancer that would not be apparent from the analysis of a single data type alone. As multi-modal data rapidly accumulate in research laboratories and public databases such as *The Cancer Genome Atlas* (TCGA), the translation of such data into clinically actionable knowledge has been slowed by the lack of computational tools capable of analyzing MMDSs. Here, we describe the *Joint Analysis of Many Matrices by ITeration* (JAMMIT) algorithm that jointly analyzes the data matrices of a MMDS using sparse matrix approximations of rank-1.

**Methods:**

The JAMMIT algorithm jointly approximates an arbitrary number of data matrices by rank-1 outer-products composed of “sparse” left-singular vectors (eigen-arrays) that are unique to each matrix and a right-singular vector (eigen-signal) that is common to all the matrices. The non-zero coefficients of the eigen-arrays identify small subsets of variables for each data type (i.e., signatures) that in aggregate, or individually, best explain a dominant eigen-signal defined on the columns of the data matrices. The approximation is specified by a single “sparsity” parameter that is selected based on false discovery rate estimated by permutation testing. Multiple signals of interest in a given MDDS are sequentially detected and modeled by iterating JAMMIT on “residual” data matrices that result from a given sparse approximation.

**Results:**

We show that JAMMIT outperforms other joint analysis algorithms in the detection of multiple signatures embedded in simulated MDDS. On real multimodal data for ovarian and liver cancer we show that JAMMIT identified multi-modal signatures that were clinically informative and enriched for cancer-related biology.

**Conclusions:**

Sparse matrix approximations of rank-1 provide a simple yet effective means of jointly reducing multiple, big data types to a small subset of variables that characterize important clinical and/or biological attributes of the bio-samples from which the data were acquired.

**Electronic supplementary material:**

The online version of this article (doi:10.1186/s13040-016-0103-7) contains supplementary material, which is available to authorized users.

## Background

Advances in array technology, high-throughput sequencing, and clinical imaging platforms enable the measurement of ten’s of thousands of variables of a specific data type in a fixed set of tissue samples [1–4]. Such “big” data types include genome-wide measurements of messenger RNA (mRNA) and microRNA expression, DNA methylation, *single nucleotide polymorphisms* (SNPs), next-generation sequence data, and quantitative features extracted from *Positron Emission Tomography* (PET) images.

The measurement of *p* > 1 variables of a given data type obtained from a collection of *n* > 1 samples can be organized into a *p* × *n* data matrix *D* with rows representing variables and columns representing measurements of the *p* variables in each of the *n* samples. For big data types we have *p* ≫ *n*, making such “tall and thin” matrices difficult to analyze using standard statistical techniques due to a severe multiple comparisons problem and low *Signal-to-Noise Ratio* (SNR) [1, 5, 6]. The low SNR is due in large part to the relatively small number of variables (out of many thousands measured) that truly represent a *Signal of Interest* (SOI) in the data that is associated with an important biological and/or clinical attribute of the samples. In this context, we are interested in selecting *s* > 0 rows of *D* that best approximate a dominant SOI in the row-space of *D* that may represent a clinically and/or biologically significant attribute of the samples. We call this subset of variables a *signature* in $D$, and if $D$ is big, then we assume that the signature is “sparse” in $D$, i.e., *s* ≪ *p*.

MMDSs pose even greater analytical challenges since the goal is to jointly analyze two or more data matrices in an integrated manner, which exacerbates problems related to data dimensionality and SNR ‘[1, 2, 7]. As before, the goal is to detect sparse signatures for each data type that individually, or in combination, explain a SOI that characterizes an important biological and/or clinical attribute of the samples. Unfortunately, the lack of analytical tools for the joint analysis of multiple data types has slowed the discovery of novel predictive biomarkers and therapeutic targets that account for interactions between networks of diverse molecular species across space and time. Falling data acquisition costs have resulted in MMDS accumulating at an exponential rate in academic research laboratories, private industry, and public data repositories such as *The Cancer Genome Atlas* (TCGA) and the *International Cancer Genome Consortium* (ICGC) [3, 8, 9]. This growing inventory of multi-modal data presents a major analytical bottleneck in the translation of big, genomic data sets into clinically actionable knowledge.

Formally, the measurements for *K* > 1 different data types collected from a common set of *n* biospecimens, *S*_*n*_ = {*ς*_1_, *ς*_2_, …, *ς*_*n*_}, can be represented by a collection of *K* data matrices, $\mathfrak{D}={\left\{{D}_k\right\}}_{k=1}^K$, where: i) *D*_*k*_ is the *p*_*k*_ × *n* data matrix representing measurements for the *k*th data type; and ii) at least one of the *D*_*k*_ is big, i.e., *p*_*k*_ > > *n*. We assume that each *D*_*k*_ has been appropriately pre-processed as function of its data type. For example, pre-processing of mRNA data would likely involve log2-transformation, quantile normalization, and row-centering, while a methylation data matrix would be transformed from Beta-values to M-values prior to normalization and row-centering [10, 11]. Following Friedland and others [12–14], let $D=D\left(\mathfrak{D}\right)$ be the *p* × *n* super-matrix that vertically “stacks” each of the pre-processed *p*_*k*_ × *n* matrices ${D}_k\in \mathfrak{D}$ along their columns where *p* = ∑_*k* = 1_^*K*^*p*_*k*_. We assume that *D* is appropriately scaled by its Frobenius norm to account for differences in the number of rows and dynamic range of the different *D*_*k*_’s. Then the joint analysis of $\mathfrak{D}$ involves the identification of *s* > 0 rows of the super-matrix *D* that models a univariate SOI in the row-space of *D* as a linear combination of the selected rows. The set of *s* variables associated with the selected rows define a *Multi-Modal SIGnature* (MMSIG) of *D* denoted by *ζ* where *s* = *dim*(*ζ*). If the SOI is highly correlated with an important biological or clinical attribute of the samples, then *ζ* explains and helps to interpret the sample attribute of interest in terms of the selected variables. Note that since *D* is big (i.e., *p* > > *n*), we want *ζ* to be sparse in *D*, (i.e., *s* ≪ *p*) to facilitate downstream interpretation and model validation. [15].

Matrix approximations of rank-1 provide an efficient way of jointly analyzing the matrices of $\mathfrak{D}$ [16–18]. For example, assume the super-matrix *D* has rank *R* > 0 and let *D* = ∑_*r* = 1_^*R*^*u*_*r*_*σ*_*r*_*v*_*r*_^*T*^ be the *Singular Value Decomposition* (SVD) of *D* where: a) *u*_*r*_ ∈ ℝ^*P*^ is the *r*th left-singular vector (i.e., the *r*th eigen-array); b) *v*_*r*_ ∈ ℝ^*n*^ is the *r*th right-singular vector (i.e., the *r*th eigen-signal); and c) *σ*_*r*_ ∈ ℝ is the *r*th singular value for *i* = 1, 2, …, *R*. Then the outer-product, *u*_1_*σ*_1_*v*_1_^*T*^, is the best rank-1 approximation of *D* in a least squares sense and *v*_1_ represents the dominant SOI on the columns of *D* that is linearly modeled in terms of the *p* rows of *D* weighted by the “loading” coefficients of *u*_1_ [16]. Let *ζ*_*SVD*_ denote the signature that selects the rows of *D* with non-zero coefficients in *u*_1_. If *D* is big, then *p* = *dim*(*ζ*_*SVD*_) is large since the SVD in general assigns a non-zero loading to each row of *D*, which poses problems for downstream validation and interpretation of *v*_1_ in terms of the *p* variables of *ζ*_*SVD*_.

**Fig. 1:**
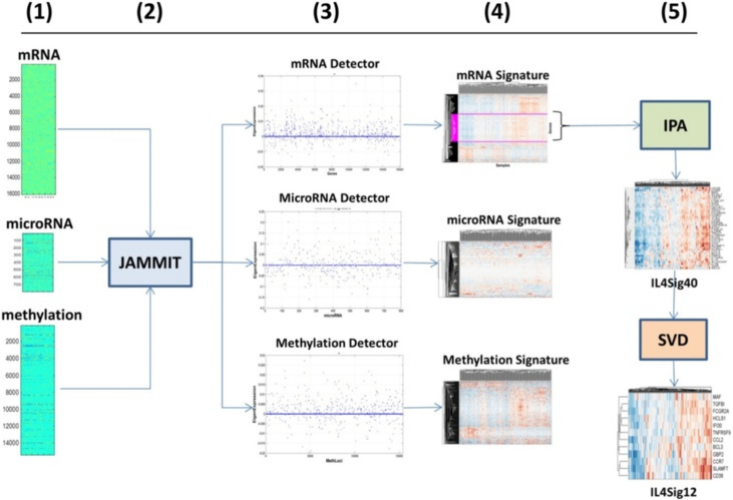
JAMMIT analysis of global mRNA, microRNA, and methylation data from 291 ovarian tumors from TCGA. This workflow focuses on iteration #1 of a JAMMIT analyses of a MMDS composed of three large data matrices that was reduced in a step-wise fashion to a 12-gene signature (see Results and discussion for more details). This mRNA signature was found to be predictive of overall survival and enriched for biology associated with immunological response in the tumor microenvironment. *Step 1*) Heat maps of mRNA, microRNA and DNA methylation data matrices assembled and pre-processed for input to JAMMIT algorithm. *Step 2*) JAMMIT analysis with minus-one cross-validation. *Step 3*) Scatter plots of sparse eigen-arrays generated by JAMMIT for each data type. Note that most of the variables for each data type have zero weighting. *Step 4*). 2-way hierarchically clustered heatmaps of each type-specific signature selected by the non-zeros coefficients of the corresponding sparse eigen-array. Note each heatmap enables the visual identification and extraction of coherent “metavariables” composed of type-specific variables that exhibit coordinated patterns of variation. *Step 5*) The mRNA meta-variable signature is further reduced using IPA and the SVD to arrive at a 12-gene expression signature that was regulated upstream by IL4. Subsequent eigene-survival and pathway analysis of the 12-gene signature established a connection between overall survival of patients with stage 3 disease being treated with platinum-based chemotherapy plus taxane and the distribution of the M1 and M2 macrophage phenotypes in the tumor micro-environment

Instead, we apply the ***BE***T ON ***S***PARSI***T***Y (BEST) principle that states that if *p* > > *n*, then it is best to assume that *v*_1_ is *sparsely* supported by a small number of rows of *D*, and employ an *ℓ*_1_ penalty to identify these rows [19]. If the sparsity assumption is true, then *v*_1_ will be optimally modeled by the selected rows; otherwise no method will be able to recover the underlying model without many more samples (i.e., Bellman’s curse of dimensionality [20].) Taking the BEST approach, we developed the *Joint Analysis of Many Matrices by ITeration* (JAMMIT) algorithm that approximates *D* by the rank-1 outer-product, *D* ≈ *uv*^*T*^, where *u* ∈ ℝ^*p*^ is a sparse eigen-array of “loading” coefficients and *v* ∈ ℝ^*n*^ is non-sparse, eigen-signal of “scores” that potentially explains an important biological and/or clinical attribute of the samples [21, 22]. The algorithm uses an “asymmetric” version of the *Least Absolute Shrinkage and Selection Operator* (LASSO) that regularizes *u* but not *v* as a function of a *ℓ*_1_ penalty term selected based on false discovery rate (FDR). The small number of non-zero coefficients of *u* define a sparse MMSIG in *D* that supports a *s*-dimensional, linear model of *v* such that *s* ≪ *p*. Since a given MMDS is likely to contain multiple SOIs of biological or clinical relevance, the JAMMIT algorithm is iteratively applied to the residuals of the current model to identify and select any additional SOI that may be present in the data (see Methods Section under The JAMMIT algorithm for more details). Figure 1 shows a specific instance of a JAMMIT analysis of three big data types for ovarian cancer downloaded from TCGA. Here, the information processing flows from left to right in five steps illustrating how three large data matrices are reduced to three relatively small type-specific signatures shown in step 4. Also shown is post-JAMMIT processing illustrating the additional pathway and matrix analysis that is needed to further reduce signature dimensionality without the loss of information. We note that the entire processing chain results in mRNA signatures that associate immune checkpoint signaling in the tumor microenvironment with response to chemotherapy.

Other methods based on matrix factorizations have been proposed for the joint analysis of multiple data types such as the *Generalized Singular Value Decomposition* (GSVD), *Joint and Individual Variation Explained* (JIVE), DISCO-SCA, *Partial Least Squares* (PLS), and *Canonical Correlation Analysis* (CCA) [12, 13, 18, 23–25]. These methods suffer from the same problem as the SVD in that they minimize the *ℓ*_2_ norm of the estimation error and assign non-zero weights to all *p* rows of *D* [26]. A number of techniques can be used to reduce the dimensionality of the selected model such as: i) rotation of principal components as implemented in factor analysis; ii) ignoring loadings smaller than some threshold; and iii) restricting the range of the loadings to a small discrete set of values [21, 27]. Unfortunately, these methods are prone to high false positive rates and poor sensitivity especially in situations where the SNR is low. Regularized versions of *Principal Components Analysis* (PCA), SVD, CCA, and PLS have been proposed for sparse signal detection and dimensionality reduction, but application of these methods to the super-matrix that “stacks” an arbitrary number of data matrices is not explicitly discussed [21, 26, 28–30]. Finally, many of the methods outlined above focus on maximal rank-*k* approximations of *D* where *k* is significantly greater than one, which precludes the use of resampling methods in the selection of the best *ℓ*_1_ penalty due to the high computational cost [30].

In what follows, we describe in greater detail a workflow for the joint analysis of multiple data types based on the JAMMIT algorithm. A section on methods provides technical detail on the algorithm and the computational tools used to evaluate the statistical significance, biological coherence, and clinical relevance of JAMMIT-derived signatures. We then present and discuss results of: 1) a study that compared JAMMIT detection performance against that of other joint analysis algorithms on simulated data; ii) a JAMMIT analysis of global mRNA, microRNA and DNA methylation data for ovarian cancer down-loaded from TCGA; and iii) a JAMMIT analysis of whole-genome mRNA data for liver cancer supervised by quantitative features derived from PET imaging data. A discussion and conclusions are presented in a final section.

## Methods

### Joint Analysis of Many Matrices by Iteration (JAMMIT)

Let *D* = {*D*_*k*_}_*k* = 1_^*K*^ denote a collection of *p*_*k*_ × *n* data matrices *D*_*k*_ that represents a MMDS acquired from a common set of *n* biospecimens, *S*_*n*_ = {*ς*_1_, *ς*_2_, …, *ς*_*n*_}. Let *D* = *stack*(*D*) denote the *p* × *n* super-matrix of *D* where *p* = ∑_*k* = 1_^*m*^*p*_*k*_. We assume that at least one *D*_*k*_ is big, so that the super-matrix *D* is also big. We assume each *D*_*k*_ has been individually pre-processed as a function of its data type as discussed in the previous section and that *D* is scaled by its Frobenius norm such that if $$ D=\left[{d}_{ij}\right] $$ is a $$ p\times n $$ matrix, then $$ D\leftarrow D\bullet / {\left\Vert D\right\Vert}_{Frob} $$ where: 1) $$ {\left\Vert D\right\Vert}_{Frob}={\left({\sum}_i\sum_j{\left|{d}_{ij}\right|}^2\right)}^{\raisebox{1ex}{$1$}\!\left/ \!\raisebox{-1ex}{$2$}\right.} $$ is the Frobenius norm of $$ D $$; and 2) $$ D/{\left\Vert D\right\Vert}_{Frob}=\left[{d}_{ij} / {\left\Vert D\right\Vert}_{Frob}\right] $$.

For *λ* > 0, the JAMMIT algorithm generates following rank-1 approximation of *D*

1 $$ D\approx u\left(\lambda \right){\left(v\left(\lambda \right)\right)}^T=u{v}^T $$

by minimizing the error function

2 $$ E\left(u,v,\lambda \right)={\left\Vert D-u{v}^T\kern0.1em \right\Vert}_{Frob}^2+\lambda {\left\Vert \kern0.1em u\kern0.1em \right\Vert}_{\ell_1} $$

subject to the constraint

3 $$ v={D}^Tu $$

where: 1) *uv*^*T*^ ∈ ℝ^*p* × *n*^ is the outer product of *u* ∈ ℝ^*p*^ and *v* ∈ ℝ^*n*^; 2) *u* is sparse relative to *p*, i.e., $s\ll p$; 3) *v* represents a SOI on the columns of *D*; 4) *λ* > 0 is an *ℓ*_1_ penalty on *u*; and 5) ${\left\Vert u\right\Vert}_{\ell_1}={\displaystyle {\sum}_{i=1}^p\left|{u}_i\right|}$ is the *ℓ*_1_ -norm of *u* ∈ ℝ^*p*^. Starting with an initial *ℓ*_2_ approximation (*u*^(0)^, *v*^(0)^) based on the SVD of $D$ such that *D* ≈ *u*^(0)^(*v*^(0)^)^*T*^, JAMMIT first obtains a *ℓ*_1_ -regularized solution vector *u*^(1)^ ∈ ℝ^*P*^ defined by

4 $$ {u}^{(1)}=\underset{u\in {\mathrm{\mathbb{R}}}^P}{ \arg \min}\left(E\left(u,{v}^{(0)},\lambda \right)\right), $$

then substitutes this solution in (3) to obtain *v*^(1)^ ∈ ℝ^*n*^ and the solution (*u*^(1)^, *v*^(1)^) that satisfies *D* = *u*^(1)^(*v*^(1)^)^*T*^. Hence, the equality constraint in Eq. (3) ensures the outer product *uv*^*T*^ in Eq. (2) represents a rank-1 approximation of *D* under the *ℓ*_1_ norm. This procedure is repeated by alternating between (2) and (3) until the sequence (*u*^(*i*)^, *v*^(*i*)^) converges to a solution (*u*, *v*) based on the error function given in (2) such that

5 $$ v={D}^Tu={\displaystyle {\sum}_{k=1}^m{D}_k^T}{u}_k. $$

Let *ζ*(*λ*) ∈ ℝ^*s*^ denote the MMSIG with non-zero entries that correspond to $s=s\left(\zeta \right)>0$ rows of *D* that support the sparse linear model in (5) as a function of *λ*. We note that: i) *λ* = 0 implies that (1) is the best rank-1 approximation of *D* based on the SVD; ii) *λ* > 0 implies that (1) is a *ℓ*_1_ -regularized, rank-1 approximation of *D* such that *s* = dim(*ζ*) ≤ *p*; and iii) there exists *λ*^sup^ > 0 such that $0\le s\le p$ if $\lambda \in \left(0,{\lambda}^{sup}\right)$. We show empirically that for simulated and real multi-modal data, one can find λ* ∈ (0, *λ*^sup^) based on an empirical estimate of FDR such that *ζ*(λ*) is sparse in *D*, i.e., *s*(λ*) = s* < < *p*.

Equation (5) suggests that parsing the vector *u* according to the order in which the *D*_*k*_’s were stacked in *D* results in individual rank-1 approximations

6 $$ {D}_k\approx {u}_k{v}^T\;\mathrm{f}\mathrm{o}\mathrm{r}\;k=1,2,\dots, m $$

where ${u}_k\in {\mathrm{\mathbb{R}}}^{s_k}$ is unique to each *D*_*k*_ and *v* represents the SOI in (1) that is shared by each *D*_*k*_. Eq. (6) implies that the MMSIG ζ* = *ζ*(λ*) = ζ*(*D*) can be similarly parsed into type-specific signatures *ζ*_k_^*^ = ζ*(*D*_*k*_) according to the stacking order of the *D*_*k*_’s in *D* that explain *v* in terms of the *k*th data type only. Moreover, we have observed empirically that the sparsity of ζ* implies that the type-specific signatures *ζ*_k_^*^ in *D*_*k*_ are also sparse if *D*_*k*_ is big. Moreover, analysis of simulated and real MMDSs show that the algorithm will still select significant rows of *D*_*k*_ even if *D*_*k*_ is not big. Table 1 outlines the key steps of a single iteration of the JAMMIT algorithm for computing joint rank-1 approximations of each ${D}_k$ of a given super-matrix *D*.

**Table 1:** JAMMIT optimization algorithm

1. Let $\mathfrak{D}=\left\{{D}_1,{D}_2,\cdots, {D}_K\right\}$ be a MMDS
2. Form pre-processed super-matrix $D= stack\left(\mathfrak{D}\right)$.
3. Compute best rank-1 approximation, $\left({u}_0,{v}_0\right)$ of *D* such that $D\approx {u}_0{v}_0^T$.
4. Compute *ℓ*_1_ -regularization ${u}_1$ of ${u}_0$: ${u}_1=\underset{u}{ \arg \min}\left({\left\Vert D-u{v}_0^T\right\Vert}_2^2+\lambda {\left\Vert u\right\Vert}_1\right)$.
5. Compute ${v}_1={D}^T{u}_1$ to obtain solution $\left({u}_1,{v}_1\right)$.
6. Assign $$ {u}_0\leftarrow {u}_1 $$ and $$ {v}_0\leftarrow {v}_1 $$.
7. Repeat steps 4–6 until convergence to final solution $\left(u,v\right)$ where $v={D}^Tu$.
8. Form MMSIG $\zeta$ composed of variables selected by the non-zero entries of $u$.
9. Parse $\zeta$ according to stacking order of the ${D}_k$ in $D$ to obtain ${\zeta}_k$ for each ${D}_k$.
10. Parse $u$ according to stacking order of ${D}_k$ in $D$ to obtain ${u}_k$ for each ${D}_k$.
11. Compute sequence of sparse rank-1 approximations $\widehat{D}=\left\{{\widehat{D}}_1,{\widehat{D}}_2,\cdots, {\widehat{D}}_K\right\}$ where ${\widehat{D}}_k\approx {u}_k{v}^T$ for $k=1,2,\cdots, K$.

Note that JAMMIT detects and models the most dominant SOI in *D* and that weaker SOI of biological and/or clinical importance could be present in *D* that are masked by the dominant SOI. Hence, we “residualize” *D* by

7 $$ {D}^{\prime }=D-u{v}^T $$

and use JAMMIT to sparsely model the most significant SOI in *D*′ [18]. This procedure is iterated until no statistically significant MMSIG are detected and modeled. In any case we hypothesize that the number of iterations is bounded by ${R}^{*}=\underset{k}{ \min}\left[ rank\left({D}_k\right)\right]$.

### Selecting an *ℓ*_1_ penalty based on false discovery rate (FDR)

For actual experimental data, empirical FDR was used to select an *ℓ*_1_ penalty that results in a MMSIG of desired size and statistical significance. Briefly, FDR was estimated for a monotone increasing sequence of *λ*’s denoted by

8 $$ \Lambda =\left\{0={\lambda}_1<{\lambda}_2<\dots <{\lambda}_l<\dots <{\lambda}_L<\infty \right\} $$

such that *λ*_1_ = 0 results in the MMSIG provided by the SVD and *λ*_*L*_ is the smallest *λ* that results in a MMSIG of length zero. The presence of statistically significant row-correlations between the matrices of *D* is indicated by a sequence of total FDR values,

9 $$ \Theta \left(\Lambda \right)=\left\{\Theta \left({\lambda}_1\right),\Theta \left({\lambda}_2\right),\dots, \Theta \left({\lambda}_{Sup}\right)\right\} $$

that decreases rapidly as a function of increasing *λ*. In this case, a λ* ∈ *Λ* can be selected such that: a) Θ(λ*) ∈ Θ(Λ) is a local minimum that is smaller than some pre-determined threshold; and b) the resulting signature, ζ* = *ζ*(λ*), is sparse in *D*. Conversely, a FDR sequence, Θ(Λ), that fails to decrease fast enough may preclude the selection of a λ* ∈ Λ that is less than a pre-determined threshold and suggests a lack of support from one or more of the ${D}_k^{\prime }s$ for the SOI. Note that a “joint” FDR sequence, Θ(Λ), can be decomposed into a collection of type-specific FDR sequences, Θ(Λ) = {Θ_*k*_(Λ)}_*k* = 1_^*K*^ based on the stacking order of the *D*_*k*_’s in *D*. Here, Θ_*k*_(Λ) represents the FDR sequence for the *k*th sub-signature, *ζ*_k_^*^ of ζ* (see Additional file 1). Again, the presence of a sparse subset of variables in *D*_*k*_ that support the common SOI in a statistically significant way is signaled by a rapidly decreasing sequence of FDR values in Θ_*k*_(Λ), while the absence of any row-support is indicated by a slowly decreasing FDR sequence, Θ_*k*_(Λ), for *k* = 1, 2, …, *K*. It follows that if all ${D}_k$ sparsely support the SOI, then all Θ_*k*_(Λ) will rapidly decrease in unison for increasing $\lambda$. Additional file 1 provides more detail on how the FDR sequences Θ(Λ) and Θ_*k*_(Λ) were generated.

### Simulated data

The detection performance of JAMMIT and other joint analysis algorithms were evaluated on 1000 simulated MMDS using Receiver Operating Characteristic (ROC) analysis (see sub-section below entitled Area under the ROC curve as a function of the *ℓ*_1_ penalty parameter). Simulated MMDS, *D*^(*η*)^ = {*D*_*k*_^(*η*)^}_*k* = 1_^2^ = {(*Σ*_*k*_^(*η*)^ + *Ν*_*k*_^(*η*)^)}_*k* = 1_^2^, for *η* = 1, 2, …, 1000 were generated where *p*_1_ and *p*_2_ were randomly selected from *P* = {1000, 2000, …, 10000}. Here, *Σ*_*k*_^(*η*)^ and *Ν*_*k*_^(*η*)^ represent simulated signal-only and noise-only data matrices, respectively, of dimensions *p*_*k*_^(*η*)^ × 50 for *k* = 1, 2 and *η* = 1, 2, …, 1000. For each *η*, the super-matrix *D*^(*η*)^ = *stack*(*D*^(*η*)^) = *Σ*^(*η*)^ + *Ν*^(*η*)^ was assembled where: 1) *p*^(*η*)^ = *p*_1_^(*η*)^ + *p*_2_^(*η*)^; 2) *Σ*^(*η*)^ = *stack*(*Σ*_1_^(*η*)^, *Σ*_2_^(*η*)^); and 3) *Ν*^(*η*)^ = *stack*(*Ν*_1_^(*η*)^, *Ν*_2_^(*η*)^).

The support of *Σ*_*k*_^(*η*)^ in *D*_*k*_^(*η*)^, denoted by *Supp*(*D*_*k*_^(*η*)^), corresponds to the non-zero components of *I*_*η*_ = *stack*(*I*_*k*_^(*η*)^(*step*), *I*_*k*_^(*η*)^(*rand*)) that identify the rows of *D*_*k*_^(*η*)^ that contain signals SS1 or SS2 defined on the 50 columns of each super-matrix *D*^(*η*)^. Here, SS1 and SS2 represent step and random functions defined on the columns of the super-matrix *D*^(*η*)^. The signal-to-noise ratio (SNR) of *D*^(*η*)^ in decibels is given by $SNR\left({D}^{\left(\eta \right)}\right)=10\times { \log}_{10}\left(\frac{\operatorname{var}\left({\overset{\frown }{\varSigma}}^{\left(\eta \right)}\right)}{\operatorname{var}\left({\overset{\frown }{N}}^{\left(\eta \right)}\right)}\right)$ where${\overset{\frown }{\varSigma}}^{\left(\eta \right)},\ {\overset{\frown }{N}}^{\left(\eta \right)}\in {\mathrm{\mathbb{R}}}^{50p}$ represent vectorized versions of *Σ*^(*η*)^ and *Ν*^(*η*)^, respectively. The goal of each simulation is to detect *Supp*(*D*^(*η*)^) such that the true positive rate is maximized for a given false positive rate over a wide range of SNR scenarios. Additional file 2 provides more detail on the generation of simulated signal-only and noise-only data matrices, *Σ*_*k*_^(*η*)^ and *Ν*_*k*_^(*η*)^, respectively, for *η* = 1, 2, …, 1000.

### Area under the ROC curve as a function of the *ℓ*_1_ penalty parameter

JAMMIT analysis of a simulated stacked matrix requires the specification of an *ℓ*_1_ penalty parameter *λ* > 0 in eq. (2), which results in a signature *ζ*(*λ*) such that *s* = dim(*ζ*(*λ*)). We note that the regularized minimization of (2) is equivalent to the un-regularized minimization of *E*(*u*, *v*) = ‖*S* − *uv*^*T*^ ‖_2_^2^ constrained by ‖*u*(*λ*)‖_1_ ≤ 1/*λ*, where the *ℓ*_1_ -parameter *λ* behaves like a threshold on the components of *u*(*λ*) ∈ ℝ^*p*^ such that larger values of *λ* result in lower-dimensional signatures [22, 31]. Hence, for a given simulated MMDS and $\lambda >0$, we can compute the sensitivity and specificity of JAMMIT to detect a signature in $D$ that supports a simulated SOI in the row-space of $D$. Consider the monotonically increasing sequence of *λ*_*k*_ ' s (denoted by $\Lambda$) defined in (8). We compute the sensitivity and specificity for each $\lambda \in \Lambda$ and plot $sensitivity$ (true positive rate) vs. $1 - \mathrm{specificity}$ (i.e., false positive rate) parameterized by $\lambda$ to generate a ROC curve. *Area Under the ROC* (AUROC) can then be used to quantify the ability of JAMMIT to detect the true support for a simulated signal embedded in a simulated super-matrix *D*. The detection performance of JAMMIT or any other detection algorithm can be compared by computing the difference between the AUROC values for JAMMIT and an alternative algorithm (ΔAUROC). A positive ΔAUROC value implies JAMMIT outperformed the alternative algorithm; otherwise the alternative algorithm outperformed JAMMIT.

### Analysis of multi-modal data for ovarian cancer downloaded from TCGA

Genome-wide mRNA, microRNA and DNA methylation data obtained from 291 tumor samples from patients with clinical stage 3 serous ovarian cancer were downloaded from TCGA (http://cancergenome.nih.gov/). This data download resulted in three high-dimensional data matrices of dimensions 16020 × 291 (mRNA), 799 × 291 (microRNA) and 15418 × 291 (DNA methylation), each of which were log-transformed, quantile-normalized, centered, and scaled by their respective Frobenius norms prior to formation of an ovarian MMDS denoted by *D*_*OVCA*_. Clinical meta-data for each patient were also downloaded from TCGA and aligned with the columns of the super-matrix of *D*_*OVCA*_. These data included censored survival time, age, stage, and treatment information. Subsequent to formation of *D*_*OVCA*_, additional whole-genome mRNA data for tumors obtained from 99 patients with Stage 3 disease were downloaded from TCGA along with associated clinical metadata. These data were organized to form a mRNA data matrix that was used to assess the robustness of any associations with overall survival with mRNA expression patterns found in the discovery data set represented by *D*_*OVCA*_.

### JAMMIT analysis of transcriptomic and PET imaging data for liver cancer

Twenty patients referred for surgical resection of liver tumors were prospectively recruited to participate in an institutional review-board approved clinical research study with written informed consent. Prior to surgery, these patients underwent liver imaging with a Philips Gemini TF-64 PET/CT scanner (Philips Healthcare, Andover, Massachusetts) using 18F-fluorocholine under an investigational new drug protocol. In a previous single-institution clinical trial, 18F-fluorocholine, a tracer of choline phospholipid synthesis, affords PET/CT with relatively high diagnostic sensitivity for HCCs [32, 33]. Presently, less is known regarding the diagnostic utility of 18F-fluorocholine for ICCs and other sub-types of liver cancer. Regions of interest (ROI) analysis of the PET/CT images were used to generate time activity curves corresponding to: 1) the arterial pool in the descending aorta; and 2) areas of tissue within the liver that corresponded to the tumor and adjacent liver samples profiled by expression arrays. PET kinetic analysis was then applied based on a *2-tissue compartment* (2TC) model of 18 F-fluorocholine pharmacokinetics in liver tumor and liver tissue [34, 35]. Pharmacokinetic parameters ${K}_1,\;{k}_2,\;{k}_3,\;{k}_4,\;{K}_1/{k}_2$, and $Flux$ for each 2TC model corresponding to each sample were estimated using PMOD 3.4 (PMOD Technologies, Zurich Switzerland) and assembled to form a $6\times 50$ Pet kinetics data matrix for the 50 tissue samples included in the experiment.

Tumor and adjacent non-tumor liver tissue specimens were obtained subsequently during surgery, and RNA was extracted from homogenized frozen tissue lysates in RLT Plus buffer with the AllPrep DNA/RNA Mini kit (Qiagen, Valencia, CA) following manufacturer’s protocol. The isolated RNA was stored at -80^0^0 until used. The quality of the total RNAs was checked on a Bioanalyzer using RNA 6000 Nano chips (Agilent, Santa Clara, CA). The RNA samples were processed following the WG-DASL assay protocol (Illumina Inc., Sunnyvale, California) and the resulting PCR products were hybridized onto the Illumina HumanHT-12 v4 Expression BeadChips included over 24,000 transcripts with genome-wide coverage of well-characterized genes, gene candidates, and splice variants. Arrays were scanned using the iScan^TM^ instrument and expression levels were quantified using GenomeStudio software (Illumina Inc., Sunnyvale, CA).

Gene-level expression values were assembled to form a 20792 × 50 data matrix where the rows represented 20792 genes and columns represented 50 adjacent-normal and tumor samples obtained from 20 patients. Here, columns 1–20 of the data matrix represented adjacent-normal samples while columns 21–50 represented 30 liver tumors of which 22 were hepatocellular carcinomas (HCCs), 6 were intra-hepatic cholangiocarcinomas (ICC) and 2 were sarcomas. The data matrix was pre-processed by generalized log2 transformation with background subtraction, quantile normalization, and row centering [36].

### Eigen-survival analysis

Let $D$ be a $p\times n$ data matrix where $p\gg n$ and let $\zeta (D)$ denote the *s* × *n* sub-matrix of $D$ composed of rows from $D$ that correspond to the variables (i.e., matrix rows) of a JAMMIT-derived signature *ζ*. Alternatively, the columns of *ζ*(*D*) can be viewed as “realizations” of the signature *ζ* in each of the *n* patients used to formulate $D$. Let *Ω*(*D*) be a 2 × *n* survival data matrix for *D* where the 1st row contains observed time-to-death for the *n* patients of *D* and the 2^nd^ row is a binary indicator of censorship for each patient (0=uncensored, 1=censored). We extracted an *Eigen-Survival Model* (ESM) based on the SVD of *ζ*(*D*) to reduce the negative impact of random noise and systematic errors on the prediction of overall survival [37, 38]. The ESM was then used to compute prognostic scores for each patient, and patients with scores in the top and bottom quartiles of scores were identified. The signature *ζ*(*D*) was predictive of survival if and only if differences in survival between patients with scores in the top and bottom quartiles were significant in both the KM and Cox regression models with *p*-value of 0.05 or less. Additional file 3 provides more detail on the workflow used to extract an ESM for a given signature.

### Ingenuity Pathway Analysis (IPA)

*Ingenuity Pathway Analysis* (IPA, QIAGEN Redwood City, California) was used to rapidly profile a given mRNA signature for enrichment in genes, canonical pathways, biological processes and upstream regulators related to cancer. In particular, IPA’s Upstream Regulator Analysis (IPA/URA) feature was used to decompose a given JAMMIT-derived signature into lower-dimensional sub-signatures composed of genes that are targeted by a single upstream regulatory molecule. In this analysis, an upstream regulator can be a chemokine, cytokine, transcription factor, drug, etc. and IPA computes an activation score and intersection *p*-value for the targeted subset of genes. The activation score measures the consistency between the observed effect of the predicted regulator on the targeted variables in our data and the predicted effect based on current knowledge as encoded in IPA. The intersection *p*-value measures the probability of a chance association between the predicted upstream regulator and its downstream targets that reside in a given signature. Note that a predicted upstream regulator does not have to be a member of the signature. Activation scores greater than 2.0 and *p*-values less than 1.0E-03 are considered significant. Signatures that are “anchored upstream” in this way inherit the function of this regulator and are thus easier to interpret biologically. IPA also generates hypotheses regarding the genes and pathways that may explain the downstream effects of a given signature on biological and disease processes.

## Results and discussion

### JAMMIT performance on simulated data

The effectiveness of JAMMIT to detect multiple signals in simulated data sets was evaluated and compared to other algorithms such the JIVE and PLS. JIVE is a generalization of *Principal Components Analysis* (PCA) to multiple data matrices. Like JAMMIT, PLS enables the supervised analysis of one matrix by another matrix and is also used for the analysis high-dimensional data sets [24]. All three algorithms were applied to the same collection of 1000 simulated MDS’s (see Methods section, Simulated Data) and tasked to detect two sparsely supported signals, SSig1 and SSig2, that were embedded in the data matrices of each simulation over a wide-range of SNR scenarios. SSig1 represents a noisy signal for differential expression that distinguishes the first 25 samples of the simulation from the last 25 samples. SSig2 on the other hand represents a random signal that is sparsely supported by rows in both data matrices that represents an unmeasured and/or unknown biological attribute of the samples.

**Fig. 2:**
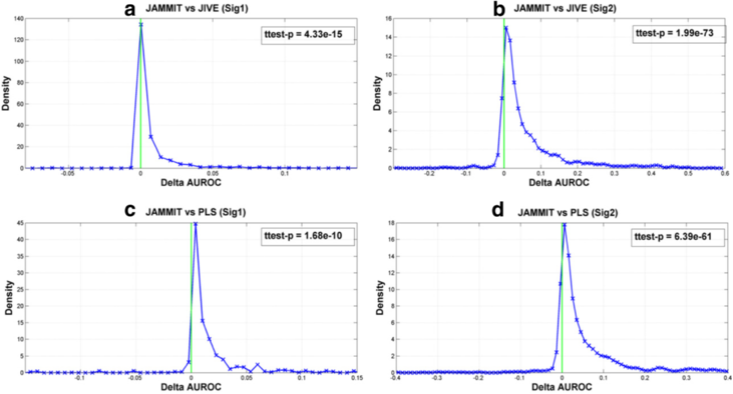
Distribution of $\Delta \mathrm{AUROC}$ values comparing JAMMIT detection performance with two other algorithms in simulated data. Panels **a** and **b** show the distributions of $\Delta \mathrm{AUROC}$ values equal to the AUROC for JAMMIT minus the AUROC for JIVE for the detection of two simulated signals, SSig1 and SSig2,  in 1000 simulated MMDS as described in the Methods section of this paper. Similarly, panels **b** and **c** show $\Delta \mathrm{AUROC}$ distributions for JAMMIT versus PLS to detect SSig1 and SSig2 in the same set of simulated MMDS used to evaluate JAMMIT versus JIVE. Each $\Delta \mathrm{AUROC}$ distribution was based on a normal kernel smoothing function evaluated at 100 equally spaces points using MATLAB’s *ksdensity* function. Note for each distribution, the area under the distribution curve is equal to one and most of this area (i.e., probability measure) is concentrated on the positive x-axis to the right of the vertical green line positioned at $x=0$. This result indicates that on average JAMMIT outperformed both JIVE and PLS in detecting the two simulated signals over a wide range of SNR scenarios

The goal of each simulation is to detect the sparse support of SSig1 and SSig2 in each simulated data matrix. Figure 2 shows distributions of ΔAUROC values that compares the ability of JAMMIT to detect the support of SSig1 and SSig2 versus that of JIVE and PLS in 1000 data simulations. For example, the first row of plots shows that the distributions of ΔAUROC values for SSig1 and SSig2 are concentrated on the positive real axis. This means that the AUROC values for JAMMIT exceeded that of JIVE more frequently than not for SSig1 and SSig2, with *p*-values of 4.33.E-15 and 1.99E-73, respectively. Similarly, the second row of plots shows that the area under the ΔAUROC distributions for both SSig1 and SSig2 is concentrated on the positive real numbers indicating that JAMMIT outperformed PLS significantly more often than not over 1000 data simulations with *p*-values of 1.68E-10 and 6.39E-61, respectively. Hence, relative to JIVE and PLS, we see that JAMMIT compares favorably in terms of ability to detect the sparse support of a step and random signal in multiple, high-dimensional data sets.

### JAMMIT analysis of ovarian cancer data from TCGA

A MMDS composed of global mRNA, microRNA and DNA methylation data obtained from 291 ovarian tumors resected from patients with stage 3 disease were downloaded from TCGA and jointly analyzed using JAMMIT. The goal was to determine if MMSIG exist that distinguished subtypes of ovarian cancer that lead to different clinical outcomes. *Leave-One-Out Cross-Validation* (LOOCV) based on JAMMIT was applied to *D* to identify a MMSIG for ovarian cancer that was robust to minus-one perturbations of the 291-sample discovery data set. First, a sequence of FDR values for a monotonically increasing sequence of *ℓ*_1_ penalty values was computed based on the JAMMIT analysis of 100 permuted versions of the super-matrix, *D* (see Methods section). An *ℓ*_1_ penalty parameter of *λ*_291_ = 0.002875.was selected based on an FDR of 0.0034619 that was a local minimum, which resulted in an mRNA signature *ζ*_*mRNA*_^(0)^ composed of 643 genes, a miRNA signature *ζ*_*miRNA*_^(0)^ composed of 368 microRNAs (FDR= 0.19912), a methylation signature *ζ*_*Meth*_^(0)^ composed of 450 methylation loci (FDR = 0.03038), and a MMSIG *ζ*^(0)^ composed of 1461 mRNA, miRNA and methylation variables that were “stacked” in the order of the *D*_*k*_’s in *D* ( FDR = 0.067647) (see Additional file 4).

For the LOOCV analysis, the *j*th column of each *D*_*k*_ of *D* was removed to obtain minus-one MMDSs, *D*^(*j*)^ = {*D*_*k*_^(*j*)^}_*k* = 1_^3^, and minus-one stacks, *D*^(*j*)^ = *stack*(*D*^(*j*)^) for *j* = 1, 2, …, 291. JAMMIT was then applied to each *D*^(*j*)^ with *λ*_291_ = 0.002875, which resulted in *s*_*j*_ -dimensional, minus-one MMSIGs, *ζ*^(*j*)^, for *j* = 1, 2, …, 291. On average, each *ζ*^(*j*)^ recapitulated 98 % of the *s*_0_ variables of *ζ*^(0)^ over all 291 minus-one analyses implying that JAMMIT-derived signatures based on *λ* = *λ*_291_ are robust to minus-one perturbations of the discovery data set. A single MMSIG defined by $\zeta =\underset{j}{\cap}\kern0.2em {\zeta}^{(j)}$ was generated, which defined sub-signatures composed of 534 mRNAs (*ζ*_1_), 337 microRNAs (*ζ*_2_) and 357 methylation loci (*ζ*_3_) common to all 291 minus-one MMSIGs.

**Fig. 3:**
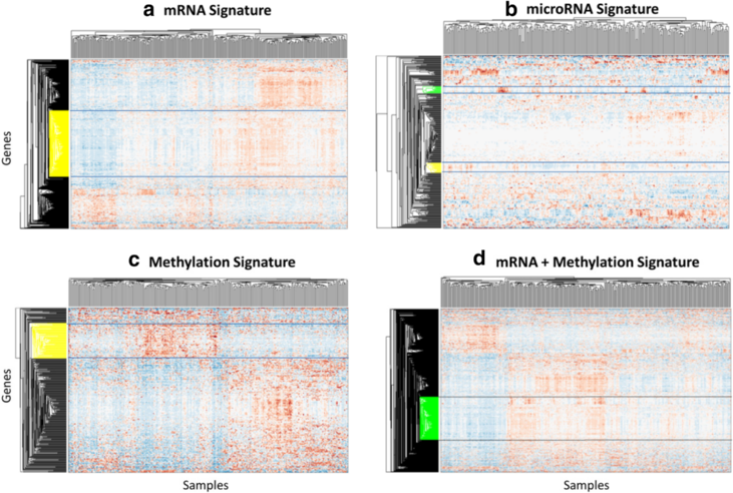
Clustered heatmaps of sparse signatures for ovarian cancer discovered by JAMMIT. **a** Heatmap of mRNA signature with one of three distinct meta-variables highlighted in yellow. **b** Heatmap of microRNA signature with two coherent meta-variables highlighted in green and yellow. **c** Heatmap of methylation signature with one of two distinct meta-variables highlighted in yellow. **d** Heatmap of joint mRNA+methylation signature with one of four distinct meta-variables highlighted in green

**Fig. 4:**
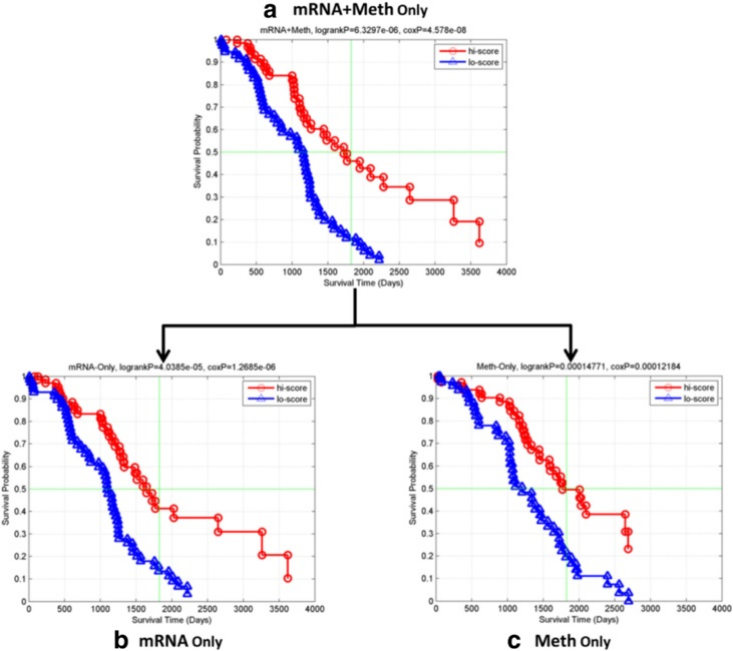
Eigen-survival analysis of JAMMIT multimodal signature composed of mRNA and methylation variables for 291 patients. **a** KM plots of based on MMSIG composed of mRNA and methylation variables. **b** KM plots based on signature composed mRNA variables only. **c** KM plots based on signature composed of methylation variables only. Note that *p*-values, median survival time and 5-year survival rate for the signature that combines variables for the mRNA and microRNA data types

Each type-specific signature obtained by JAMMIT was analyzed individually and in various combinations using hierarchical cluster analysis to identify “metagenes”, i.e., subsets of variables that exhibited coordinated, low-frequency variation of expression over the 291 samples of the discovery data set. Such coherent variation offers the best opportunity to identify novel, low-dimensional signatures that capture important biological and/or clinical attributes of the tumor samples. Figure 3 shows hierarchically clustered heatmaps of the three type-specific signatures, *ζ*_1_, *ζ*_2_, and *ζ*_3_, for mRNA, microRNA and methylation, respectively, and a MMSIG, *ζ*_13_, that “stacked’ the mRNA and methylation signature. Here, the subscript “13” denotes the concatenation of the mRNA (1) and methylation (3) signatures derived by JAMMIT. This particular combination was chosen because the FDR values for *ζ*_1_^(0)^ and *ζ*_3_^(0)^ were highly significant compared to *ζ*_2_^(0)^, which implied the type-specific signatures *ζ*_1_ and *ζ*_3_ best explained the common SOI shared by all three different data types. Visual examination of Fig. 3a-c shows that the clustered heatmaps for each type-specific signature contained meta-variables composed of matrix rows that exhibited coordinated patterns of variation, some of which are highlighted in yellow or green. In particular, the clustered heatmap for *ζ*_13_ in Fig. 3d contained the metagene, *γ*, (highlighted in green) that defined a MMSIG composed of 249 variables of which 209 were mRNAs (*γ*_1_), and 40 were methylation loci (*γ*_3_). Figure 4 shows that the MMSIG, *γ*, and the type-specific sub-signatures, *γ*_1_, and *γ*_3_ were all significantly associated with overall survival on the 291 discovery samples contained in *S*_*n*_. Interestingly, the signature that combined the mRNA and methylation variables had a more significant association with survival than signatures that contained only mRNA or only methylation variables based on log-rank and Cox regression *p*-values, median survival time, and 5-year survival rate.

**Fig. 5:**
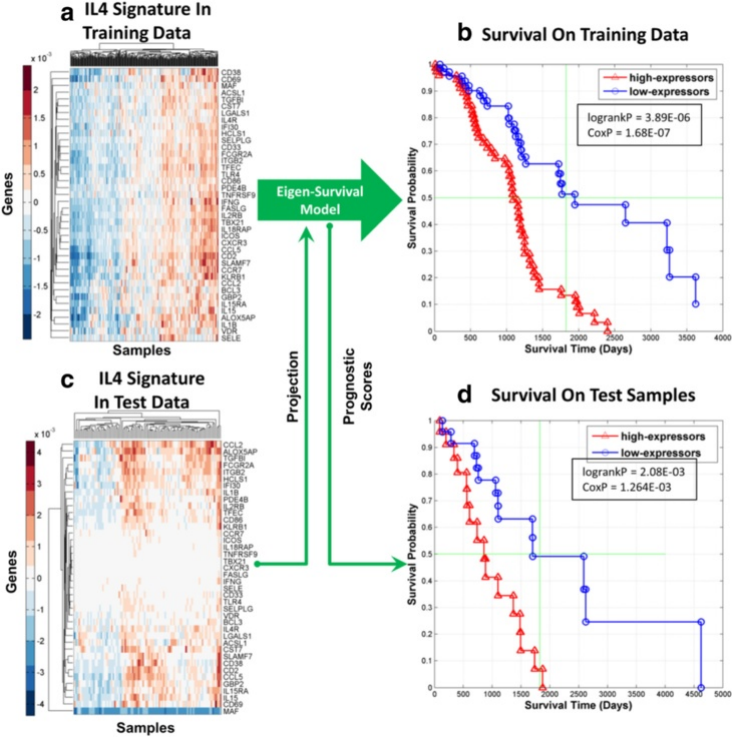
40-gene signature *φ*_*IL*4_^(40)^ for ovarian cancer anchored upstream by IL4 is robustly associated with survival. **a** Clustered heatmap of the mRNA signature *φ*_*IL*4_^(40)^ realized in the 291-sample training data set. **b** KM plots of patients in training data set with prognostic scores in the top and bottom quartiles based on the eigen-survival model based on the realization of *φ*_*IL*4_^(40)^ in 291-sample discovery data set. **c** Clustered heatmap of *φ*_*IL*4_^(40)^ realized in the 99-sample test data set. **d** KM plots of patients in unseen test data set with prognostic scores in the top and bottom quartiles. The prognostic scores for the test patients were obtained by projecting the realization of *φ*_*IL*4_^(40)^ in the test data onto the eigen-survival model for *φ*_*IL*4_^(40)^ derived from the discovery data set (green arrows)

To further reduce signature dimensionality and to better understand the biology that underlay the association of *γ* with overall survival, we focused subsequent downstream analysis and interpretation on the 209-gene mRNA signature, *γ*_1_, using IPA. In particular, the Upstream Regulator Analysis (URA) feature in IPA was used to identify sub-signatures of *γ*_1_ that were “anchored” upstream by a single regulating molecule. Table 2 shows that Interleukin 4 (IL4) was the top upstream regulator of *γ*_1_ that directly targeted 40 genes (out of 209) in the signature (Score=2.115 p=2.11E-20). Note that activation scores greater than 2.0 and *p*-values less than 1.0E-03 are considered significant. The 40 genes in *γ*_1_ directly targeted by IL4 were used to define a mRNA signature *φ*_*IL*4_^(40)^ contained in *γ*_1_ that was “anchored” upstream by IL4. Figure 5 shows the results of an eigen-survival analysis based on the realization of *φ*_*IL*4_^(40)^ in the expression data for the 291 patients in the discovery data set. Figure 5a shows the clustered heatmatp of *φ*_*IL*4_^(40)^ realized in the training data set and Fig. 5b shows KM plots based on prognostic scores for each patient derived from the ESM extracted from the expression patterns in Fig. 5a. In Fig. 5b, we see that 144 patients with prognostic scores in the top and bottom quartiles have significantly different KM plots with log-rank *p*-value of 3.89E-06 (logrankP). Moreover, a Cox regression model of overall survival based on prognostic scores for all 291 patients with age as a covariate had a *p*-value of 1.68E-07 (CoxP), which provides further validation of the eigen-survival model derived from expression patterns visualized in Fig. 5a. Figure 5c shows the clustered heatmap of the *φ*_*IL*4_^(40)^ signature realized in whole-genome mRNA data for 99 independent test tumor samples. The prognostic scores for the 99 test patients were computed by processing the expression patterns in Fig. 5c using the ESM derived from the expression patterns in Fig. 5a. Figure 5d shows that test patients with prognostic scores in the top and bottom quartiles have significantly different survival statistics (logrankP=2.08E-03, CoxP=1.26E-03). Hence, the ESM based on *φ*_*IL*4_^(40)^ captured information related to overall survival that was also applicable to the 99-samples of the independent test data set that were unseen during discovery.

**Table 2:** Top Upstream Regulators of mRNA signature ${\gamma}_1$ for ovarian cancer

Upstream regulator	Predicted state	Activation score	Intersection *P*-value	Number of targets
IL4	Activated	2.115	2.115E-20	40
OSM	Activated	2.616	2.41E-08	21
Stat5(A/B)	Activated	2.630	6.50E-08	9

IPA identified IL4 as the top upstream regulator of the ${\boldsymbol{\gamma}}_1$ signature that directly targeted 40 genes in the signature (Score=2.115, p=2.115E-20). These 40 genes formed a mRNA signature, *φ*_*IL*4_^(40)^, that was “anchored” upstream by IL4 with expression patterns that implied the up-regulation of this gene. Subsequent eigen-survival analysis shows that the *φ*_*IL*4_^(40)^ signature was robustly associated with overall survival on the 291-sample discovery data set and a 99-sample independent test data set. Regulation of *φ*_*IL*4_^(40)^ by IL4 linked overall survival of ovarian cancer patients with stage 3 disease to macrophage polarization in the tumor environment

**Fig. 6:**
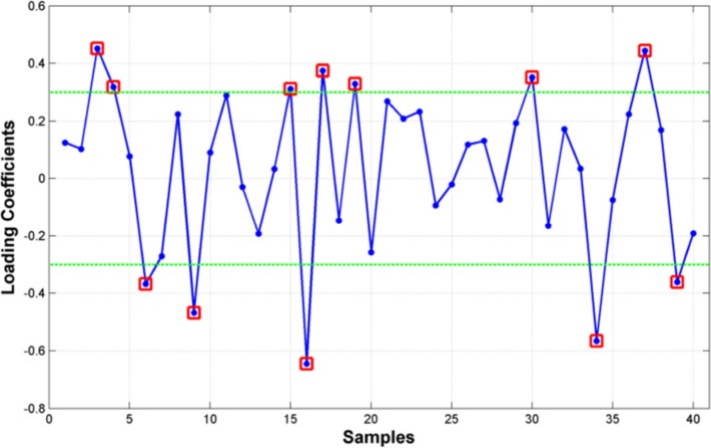
Loading coefficients of eigen-survival model derived from *φ*_*IL*4_^(40)^ in the 291-sample discovery data set. Genes that contribute most significantly to the eigen-survival model derived from the 291-sample discovery data set are highlighted by red squares. These genes were used to define a 12-gene mRNA signature *φ*_*IL*4_^(12)^ that was evaluated for association with overall survival and biological coherence

**Fig. 7:**
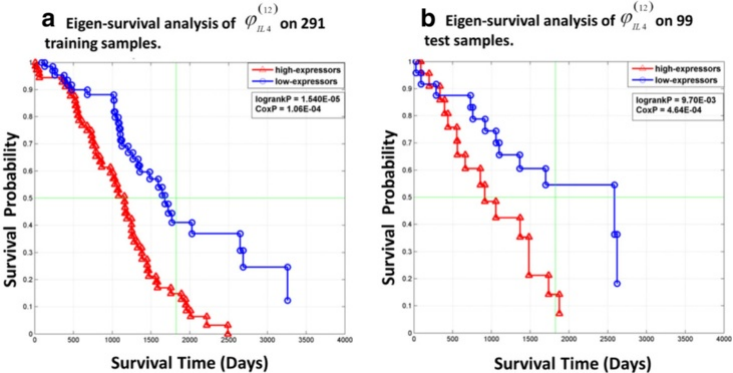
A 12-gene mRNA signature for ovarian cancer anchored upstream by IL4 predicts overall survival. **a** KM plots of patients in discovery data set with prognostic scores based on the 12-gene mRNA signature *φ*_*IL*4_^(12)^ in the top (red) and bottom (blue) quartiles. Note the two groups of 72 patients each (144 total) show significant differences in survival based on the separation between their respective KM plots. **b** KM plots of patients in test data set with *φ*_*IL*4_^(12)^ prognostic scores in the top (red) and bottom (blue) quartiles. The two groups of 24 patients each (48 total) show significant differences in survival based on the separation between their respective KM plots. Note the prognostic scores for the test patients were computed by projecting the test data matrix for *φ*_*IL*4_^(12)^ onto the ESM derived from discovery data matrix for *φ*_*IL*4_^(12)^

We further reduced the dimensionality of *φ*_*IL*4_^(40)^ based on the ESM extracted from the 291 discovery samples. Figure 6 shows a plot of the 40 loading coefficients associated with the ESM derived from expression patterns in Fig. 5a with 12 high magnitude coefficients highlighted in red. The 12 genes corresponding to these coefficients were assembled to form the mRNA signature, *φ*_*IL*4_^(12)^, that was tested for association with overall survival on the 291-sample discovery data set and the 99-sample independent test data set. Figure 7a shows that ESM based on *φ*_*IL*4_^(12)^ in the 291 samples of the discovery data set was significantly associated with overall survival (logrankP=1.54E-05, CoxP=1.06E-04). Moreover, Fig. 7b shows that the ESM based on *φ*_*IL*4_^(12)^ realized in the discovery data generalizes to the 99 samples of the independent test data set (logrankP=9.70E-03, CoxP=4.64E-04). Interestingly, the set of 28 genes in *φ*_*IL*4_^(40)^ complementary to the genes in *φ*_*IL*4_^(12)^ failed to generalize on the 99 independent test samples. These results validate the BEST principle as implemented by JAMMIT for the joint analysis of multiple data sets in ovarian cancer.

Note that IL4 directly targets every gene in *φ*_*IL*4_^(12)^ per IPA. IL4 induces the transformation of Tumor Associated Macrophages (TAMs) that infiltrate the tumor microenvironment into the M2 phenotype, which confers a survival advantage to cancer cells and promotes tumor growth [39, 40]. An alternative pathway involving Interferon Gamma (IFNG) and Tumor Necrosis Factor Alpha (TNFA) transform TAMs into the M1 phenotype that exerts a cytotoxic effect on genetically mutated cancer cells. It has been reported that a high M1/M2 ratio is associated with extended survival in ovarian cancer patients [39]. This suggests that immune cell polarization in the tumor microenvironment impacts the overall survival of patients with ovarian cancer undergoing standard platinum-based chemotherapy combined with paclitaxel. Indeed, the *φ*_*IL*4_^(12)^ signature contains the Chemokine (C-C motif) Ligand 2 (CCL2) gene, which is a chemokine that recruits monocytes from the bloodstream to the tumor microenvironment [41]. It has been reported that CCL2 is up-regulated in ovarian cancer and the blockade of CCL2 protein expression enhances immunotherapeutic and chemotherapeutic response [41].

### Imaging-genomics of liver cancer

Whole-genome expression data were collected for 20792 genes in 20 adjacent-normal, 22 hepatocellular carcinoma (HCC), 6 intra-hepatic cholangiocarcinoma (ICC) and 2 sarcoma samples using DASL microarrays. The expression data were assembled to form a 20792 × 50 expression data matrix where columns 1–20 represented the normal samples and columns 21–50 represented the tumor samples. The data matrix of raw expression was pre-processed by generalized log2 transformation, quantile normalization, and row-centering to obtain the pre-processed expression data matrix *H*_*mRNA*_. The values of six kinetic parameters, ${K}_1,\;{k}_2,\;{k}_3,\;{k}_4,\;{K}_1/{k}_2,\; Flux$ obtained from 2TC models for each tissue sample formed the columns of a 6 × 50 data matrix that was row-centered to obtain the PET data matrix, *H*_*PET*_. A final pre-processing step involved the scaling of the stacked matrix *H*_*PETX*_ = *stack*(*H*_*mRNA*_, *H*_*PET*_) by its Frobenius norm. The goal of this analysis is to identify mRNA signatures that are highly correlated with the rows of the PET kinetic data matrix [42, 43].

Six different analyses of *H*_*mRNA*_ based on JAMMIT were conducted where each analysis was supervised by a single PET kinetic parameter. That is, JAMMIT was applied to *H*_*PETX*_^(*l*)^ = {*H*_*mRNA*_, *H*_*PET*_^(*l*)^} where *H*_*PETX*_^(*l*)^ is a 1-dimensional vector equal to the $l$ th row of *H*_*PET*_ for *l* = 1, 2, …, 6. Of the six possible analyses, only supervision by the ${H}_{PETX}^{(5)}={K}_1/{k}_2$ kinetic parameter resulted in a FDR profile that implied significant joint correlations between *H*_*mRNA*_ and *H*_*PET*_ (see Additional file 5). A locally minimal FDR* = 0.000549 was selected from the FDR profile for genes that corresponded to an ${\ell}_1$ penalty parameter value of λ* = 0.0089429. A JAMMIT analysis based on this value of *λ* resulted in a mRNA signature ${\omega}_{mRNA}^{\left({K}_1/{k}_2\right)}$ containing 652 genes that was significantly correlated with the ${K}_1/{k}_2$ kinetic parameter. Persistently low FDR values for ${\omega}_{mRNA}^{\left({K}_1/{k}_2\right)}$ as a function of *λ* implied a significant and robust correlation between ${\omega}_{mRNA}^{\left({K}_1/{k}_2\right)}$ and the ${K}_1/{k}_2$ PET parameter over a wide-range of sparse, linear models. Moreover, the dominant eigen-signal of the 652 × 50 signature matrix, ${\omega}_{mRNA}^{\left({K}_1/{k}_2\right)}\left({H}_{mRNA}\right)$ was significantly correlated with the ${K}_1/{k}_2$ PET parameter ($r=0.413,\;p=0.00293\Big)$. In sharp contrast, the FDR profiles for JAMMIT analyses of *H*_*mRNA*_ supervised by the other PET kinetic parameters failed to produce an *ℓ*_1_ penalty that correlated the two data types (see Additional file 6). Note these results show that JAMMIT is able to identify significant variables of data types defined by a small number of variables. Indeed, the data matrix *H*_*mRNA*_ described above has 20792 rows, while the PET kinetic data matrix, ${H}_{PETX}^{(5)}$, has a single row composed of ${K}_1/{k}_2$ kinetic parameter values in 50 samples. Here, the FDR table for the joint analysis of *H*_*mRNA*_ and ${H}_{PETX}^{(5)}$ admits the single row of ${H}_{PETX}^{(5)}$ into the sparse, rank-1 approximation of *D*_*PETX*_^(*l*)^ = *stack*{*H*_*mRNA*_, *H*_*PET*_^(*l*)^} for almost all *ℓ*_1_ parameter values (see Additional files 5 and 6).

**Fig. 8:**
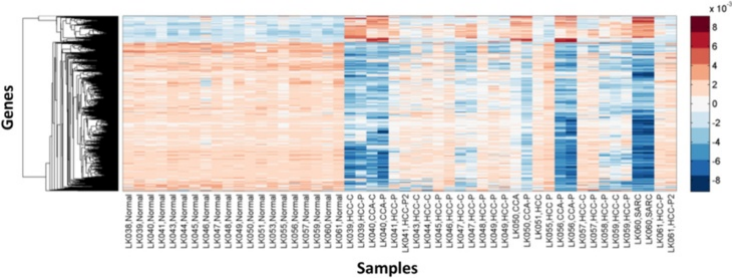
Clustered heatmap of the *K*_1_/*k*_2_ signature identified by JAMMIT in 50 liver tissue samples. The heatmap for the *K*_1_/*k*_2_ signature, ${\omega}_{mRNA}^{\left({K}_1/{k}_2\right)}$, exhibits very uniform expression on the normals (columsn 1–20) and very high variability on the tumor samples. On the tumor samples, we note significant down-regulation of ${\omega}_{mRNA}^{\left({K}_1/{k}_2\right)}$ expression patterns on a subset of seven (7) HCC, six (6) ICC and 2 sarcomas. The remaining 15 HCC had ${\omega}_{mRNA}^{\left({K}_1/{k}_2\right)}$ expression profiles very similar to the 20 normal samples

**Fig. 9:**
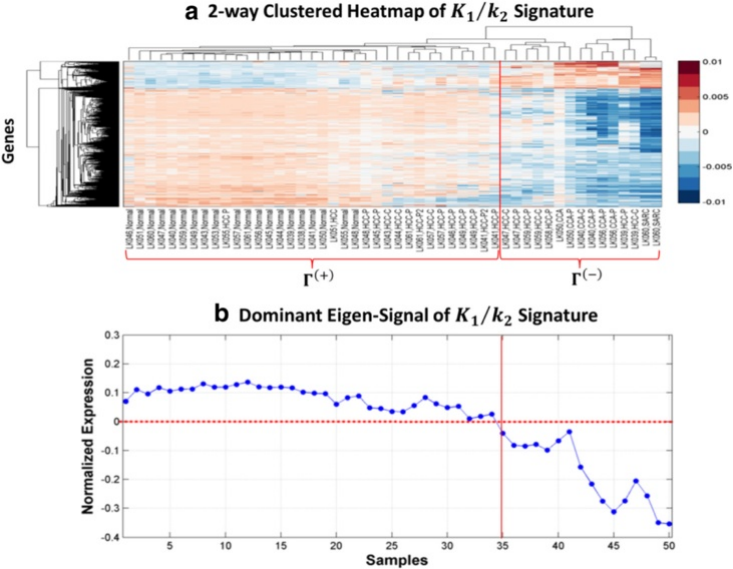
Cluster analysis by the *K*_1_/*k*_2_ signature reveals a novel subtype of HCC metabolically similar to ICC. **a** 2-way hierarchically clustered heatmap of *K*_1_/*k*_2_ signature in the 50-sample discovery data set. This analysis identified two distinct expression phenotypes *Γ* ^(−)^ and *Γ* ^(+)^ where *Γ* ^(−)^ included samples where ${\omega}_{mRNA}^{K_1/{k}_2}$ was down-regulated on the samples in *Γ* ^(−)^ relative to the remaining samples in *Γ* ^(+)^. The *Γ* ^(−)^ class contained all 6 ICC samples plus 7 HCC and 2 sarcomas while *Γ* ^(+)^ contained all 20 normal samples along with 15 HCC. **b** Plot of the dominant eigen-signal of the matrix for the ${\omega}_{mRNA}^{K_1/{k}_2}$ signature clearly separates the samples in *Γ* ^(−)^ and *Γ* ^(+)^ based on a threshold set at zero

Figure 8 visualizes the realization of ${\omega}_{mRNA}^{\left({K}_1/{k}_2\right)}$ in *H*_*mRNA*_ as a row-clustered heatmap where we see that aggregate gene expression is highly variable on the tumor samples (columns 21–50) compared to the normal samples (columns 1–20). Figure 9a shows a 2-way clustered heatmap of ${\omega}_{mRNA}^{\left({K}_1/{k}_2\right)}$ and here we see a group of genes in ${\omega}_{mRNA}^{\left({K}_1/{k}_2\right)}$ that are preferentially down-regulated on a set of 15 tumors relative to a complementary subset of fifteen (15) HCCs and twenty (20) normal samples. Let *Γ*^(−)^ denote the set of column indices of *H*_*mRNA*_ that correspond to the samples where ${\omega}_{mRNA}^{\left({K}_1/{k}_2\right)}$ is down-regulated and *Γ*^(+)^ column indices for samples where ${\omega}_{mRNA}^{\left({K}_1/{k}_2\right)}$ is up-regulated. In Fig. 9b we see that the dominant eigen-signal of the 2-way, clustered heatmap in Fig. 9a clearly discriminates between the samples in *Γ*^(−)^ and *Γ*^(+)^ based on a threshold set at zero. The ability of ${\omega}_{mRNA}^{\left({K}_1/{k}_2\right)}$ to discriminate between the samples in *Γ*^(−)^ and *Γ*^(+)^ suggests two distinct expression phenotypes for HCC represented by the seven (7) HCC in *Γ*^(−)^ and fifteen (15) HCC in *Γ*^(+)^. Moreover, the co-clustering of 7 HCC samples in *Γ*^(−)^ along with 6 ICC suggests that these HCC samples represent a cholangio-like HCC subtype (CL-HCC), which may share clinical and biological attributes of this more aggressive sub-type of liver cancer [44, 45].

Table 3 lists the top canonical pathways and upstream regulators of ${\omega}_{mRNA}^{\left({K}_1/{k}_2\right)}$ according to IPA. The top upstream regulators included the nuclear receptors HNF4A, HNF1A, and FXR (NR1H4) where HNF4A and HNF1A were predicted to be inactivated with high statistical significance. Moreover, FXR/LXR and LXR/RXR Activation were the top canonical pathways and most of the genes in both pathways were down-regulated suggesting inactivation of these pathways upstream of ${\omega}_{mRNA}^{\left({K}_1/{k}_2\right)}$. The dominate downstream effects of ${\omega}_{mRNA}^{\left({K}_1/{k}_2\right)}$ per IPA included biological functions related to the dysregulation of lipid and bile acid metabolism as well as disease functions related to the initiation and progression of HCC and ICC. For example, the inactivation of HNF4A as a significant upstream regulator of ${\omega}_{mRNA}^{\left({K}_1/{k}_2\right)}$ is consistent with published reports that HNF4A down-regulation suppresses hepatocyte differentiation and commitment to the biliary lineage in ICC and CL-HCC [44–47]. Moreover, loss of HNF1A function in hepatocytes leads to the activation of pathways involved in tumorigenesis [48]. Finally, HNF4A and FXR exhibit reduced expression in human HCC and ICC, and that mice lacking FXR expression spontaneously developed HCC [49–51].

**Table 3:** IPA analysis identifies top canonical pathways and upstream regulators of the ${\omega}_{mRNA}^{K_1/{k}_2}$ signature for liver cancer

Top Canonical Pathways		
Pathway	*P*-Value	Overlap
FXR/RXR Activation	3.03E-60	48.8 % (62/127)
LXR/RXR Activation	2.36E-37	37.2 % (45/121)
LPS/IL1 Mediated Inhibition of RXR Function	5.89E-25	20.5 (45/219)
Top Upstream Regulators		
Upstream Regulator	*P*-Value of Overlap	Predicted Activation
HNF1A	2.02E-78	Inhibited
PPARA	4.40E-46	
HNF4A	4.20E-44	Inhibited
FXR	1.95E-38	
GW4064	1.85E-34	Inhibited

The ${\omega}_{mRNA}^{K_1/{k}_2}$ signature was highly enriched for genes in the FXR/RXR Activation pathway according to IPA. This pathway regulates lipid and bile acid metabolism and has been associated with the initiation and progression of liver cancer. The top upstream regulators of ${\omega}_{mRNA}^{K_1/{k}_2}$ are the nuclear receptors HNF1A, HNF4A and FXR that are known regulators of membrane transport function and have also been associated with liver cancer

We note the ${\omega}_{mRNA}^{\left({K}_1/{k}_2\right)}$ signature included 46 membrane transport genes from the *ATP-Binding Cassette* (ABC) and S*olute Carrier* (SLC) super-families, almost all of which were significantly down-regulated in the tumor samples of *Γ*^(−)^ relative to the samples in *Γ*^(+)^. Recall the dominant eigen-signal of ${\omega}_{mRNA}^{\left({K}_1/{k}_2\right)}\left({D}_1\right)$ was found to be significantly correlated with the *K*_1_/*k*_2_ PET parameter ($r=0.413,\;p=0.00293\Big)$. The *K*_1_/*k*_2_ parameter in-fluorocholine PET images reflects the blood-tissue equilibrium of choline, a nutrient important for phospholipid and bile homeostasis, as well as lipid transform. Therefore, it is not surprising that the ${\omega}_{mRNA}^{\left({K}_1/{k}_2\right)}$ signature contained a significant number of ABC and SLC membrane transport genes, since these genes regulate the influx and efflux of bile and lipids across the membranes of hepatocytes and cholangiocytes and are tightly regulated by nuclear receptors HNF4A, HNF1A and FXR [52]. The above suggests the inactivation of HNF4A, HNF1A and FXR upstream of ${\omega}_{mRNA}^{\left({K}_1/{k}_2\right)}$ suppresses the uptake and efflux of bile and lipids downstream of ${\omega}_{mRNA}^{\left({K}_1/{k}_2\right)}$ by down-regulating the expression of specific ABC and SLC genes of ${\omega}_{mRNA}^{\left({K}_1/{k}_2\right)}$. In addition to the wide-spread disruption of bile acid and lipid homeostasis, the down-regulation of membrane transporters in ${\omega}_{mRNA}^{\left({K}_1/{k}_2\right)}$ directly impacts liver carcinogenesis and tumor progression. For example: i) SLC22A1 is associated with progression and survival in human ICC [53]; ii) knockout mice lacking ABCB4 suffer from the loss of biliary phospholipid secretion and spontaneously develop HCC [50]; iii) transporter genes ABCB1, ABCC6, ABCC9, ABCG2 are down-regulated in prostate cancer [54]; iv) ABCB11/BSEP (Bile Salt Export Pump) and FXR expression is reduced in HCC [55]; and v) SLC22A1 is epigenetically silenced in human HCC [56].

**Fig. 10:**
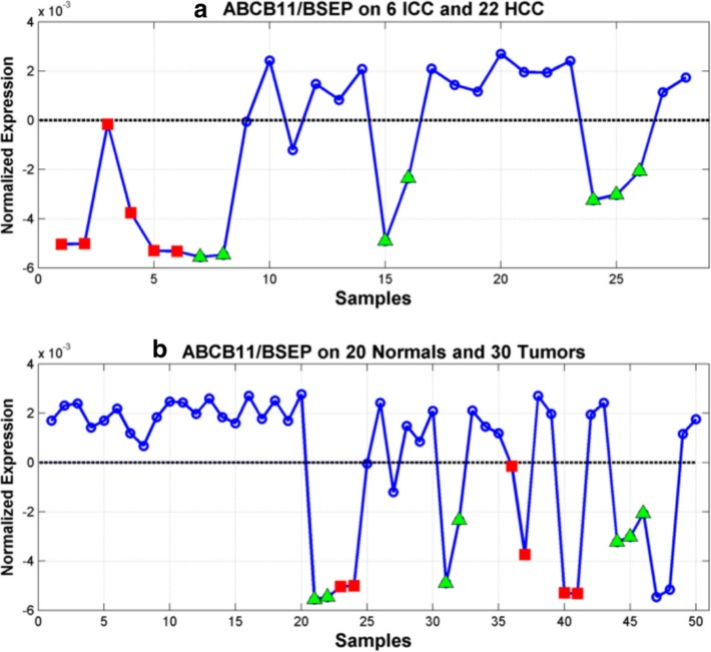
The ABCB11 gene discriminates between the *Γ* ^(−)^ and *Γ* ^(+)^ expression phenotypes. **a** ABCB11 expression over 6 ICC (columns 1–6) and 22 HCC (columns 7–28). **b** ABCB11 expession over 20 normals (columns 1–20) and 30 tumors (columns 21–50)

Figure 10 shows the expression profiles of the ABCB11 gene (i.e., Bile Salt Export Pump or BSEP), in two different groupings of the samples: i) ICCvsHCC compares 6 ICC (columns 1–6) and 22 HCC (columns 7–28); and ii) NRMvsTUMOR compares 20 Normals (columns 1–20) and 30 Tumors (columns 21–50). The top panel of Fig. 10 shows that the ABCB11 gene is down-regulated in the ICC samples (red squares) and CL-HCC samples (green triangles) relative to the HCC samples (blue circles) in the ICCvsHCC data set based on a horizontal threshold set at zero. The bottom panel of Fig. 10 shows that ABCB11 is uniformly up-regulated on the 20 normals and highly variable on the tumors with preferential down-regulated on the ICC (red circles), CL-HCC (green triangles) and sarcoma samples in the NRMvsTUMOR data set. The ABCB11 gene codes for a protein that facilitates the efflux of bile acids out of the liver and defects in the ABCB11 gene result in progressive familial intrahepatic cholestasis, which is a progressive liver disease that often starts early in life and rapidly progresses to end-stage liver disease with an increased risk for HCC. The above suggests that ICC and CL-HCC subtypes can be characterized in part by the suppression of bile acid efflux that is mediated by the down-regulation of the ABCB11 transporter gene.

**Fig. 11:**
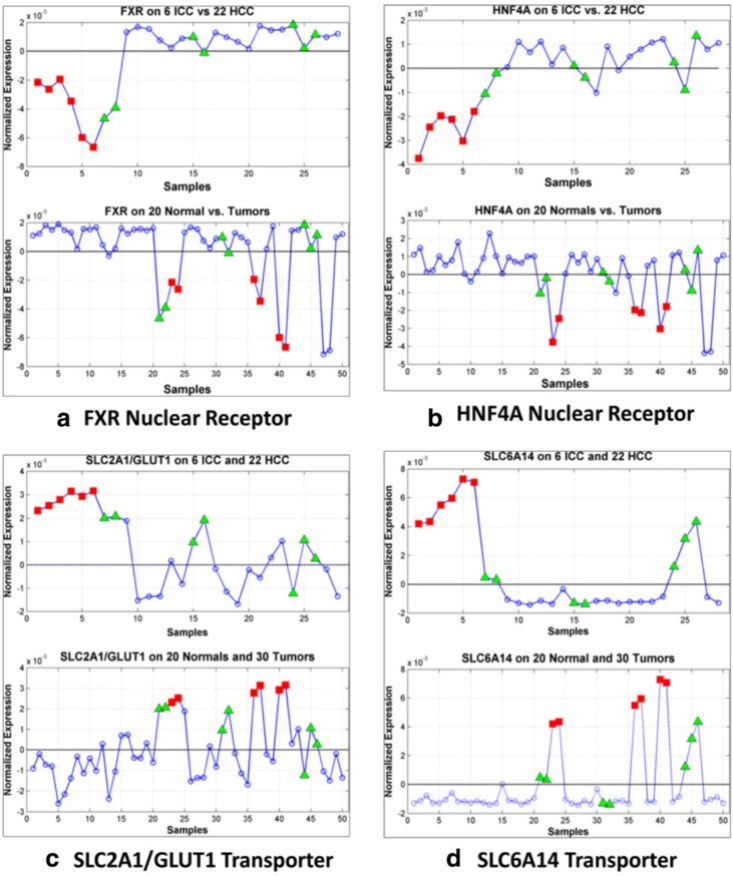
Expression profiles of selected nuclear receptors and transporter genes associated with the ${\boldsymbol{K}}_1/{\boldsymbol{k}}_2$ liver signature. Shown are normalized expression profiles of selected genes associated with the ${\boldsymbol{K}}_1/{\boldsymbol{k}}_2$ signature in two experimental designs denoted by ICCvsHCC and NRMvsTUMOR. Each lettered panel contains top and bottom sub-panels showing the profile of a gene in the ICCvsHCC and NRMvsTUMOR designs, respectively. In the top panels, columns 1–6 represent ICC samples and columns 7–28 HCC samples, while in bottom sub-panels, columns 1–20 represent normal samples and columns 21–50 represent liver tumors (6 ICC, 2 sarcomas and 22 HCC). Red squares represent ICC samples, green triangles represent CL-HCC samples, and blue circles represent normal and HCC samples. **a** Top panel shows FXR is down-regulated on ICC (cols 1–6) relative to HCC while the bottom panel shows that FXR is uniformly up-regulated on the normals and preferentially down-regulated on a subset of tumors that includes 6 ICC and 2 of 7 CL-HCC. **b** HNF4A shows expression patterns similar to FXR over the two groupings of the samples, i.e., preferential down-regulation on the ICC and CL-HCC relative to the normals and HCCs. **c** SLC2A1/GLUT1 is a transporter that is negatively correlated with the ${\boldsymbol{K}}_1/{\boldsymbol{k}}_2$ PET parameter and preferentially up-regulated on the ICC and CL-HCC samples relative to the normal and HCC samples. **d** SLC6A14 is strikingly up-regulated on all 6 ICC samples and less so on 5 of 7 CL-HCC samples relative to the normal and HCC samples

Figure 11 shows the expression profiles of nuclear receptors FXR and HNF4A and the SLC transporter genes SLC2A1/GLUT1 and SLC6A14 in the ICCvsHCC and NRMvsTUMOR experiments. Panels A and B of Fig. 11 confirm that both FXR and HNF4A are preferentially down-regulated in ICCs relative to the HCC, uniformly up-regulated on the normals relative to liver tumors, and highly variable on the tumors with preferential down-regulation on the tumors in *Γ*^(−)^. Panel C of Fig. 11 shows that unlike the nuclear receptors FXR and HNF4A, the SLC2A1/GLUT1 transporter is up-regulated in ICC relative to HCC, uniformly down-regulated on normals relative to tumors, and highly variable on tumors but with preferential up-regulation on the tumors in *Γ*^(−)^. In Fig. 11d, SLC6A14 shows strikingly high and specific up-regulation on all 6 ICC and 5 of 7 CL-HCC samples relative to the remaining 15 HCC samples in the ICCvsHCC experimental. Moreover, we see that SLC6A14 is uniformly down-regulated on the normals compared to the tumors in NRMvsTUMOR with significant up-regulation concentrated on the ICC and CL-HCC samples. SLC6A14 is reported to be highly activated in cancers of the colon, cervix, breast, and pancreas, and the blockade of SLC6A14 has been suggested as a treatment for many solid tumors [57, 58]. The expression profiles in Fig. 11d supports the possibility that SLC6A14 may be a therapeutic target ICC and CL-HCC.

**Fig. 12:**
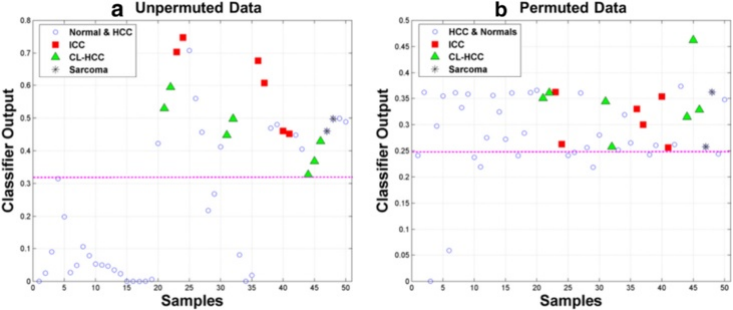
Discriminating between two expression phenotypes based on the PET kinetic parameter ${\boldsymbol{K}}_1/{\boldsymbol{k}}_2$. Points in scatter plots represent output of Generalized Regression Neural Networks (GRNNs) trained to discriminate between two expression phenotypes denoted by ${\boldsymbol{\Gamma}}^{\left(-\right)}$ and ${\boldsymbol{\Gamma}}^{\left(+\right)}$ identified by the ${\boldsymbol{\omega}}_{\boldsymbol{mRNA}}^{\left({\boldsymbol{K}}_1/{\boldsymbol{k}}_2\right)}$ expression signature. Expression phenotype ${\boldsymbol{\Gamma}}^{\left(-\right)}$ contains 7 HCC, 6 ICC and 2 sarcomas while phenotype ${\boldsymbol{\Gamma}}^{\left(+\right)}$ contains 20 normals and 25 HCC. In each panel, columns 1–20 represent normals and columns 21–50 represent liver tumors (15 HCC, 6 ICC, 2 sarcomas, 7 CL-HCC). Horizontal line (magenta) represents a threshold $\boldsymbol{\tau}$ on the GRNN output where samples with GRNN values greater than $\boldsymbol{\tau}$ are assigned to ${\boldsymbol{\Gamma}}^{\left(-\right)}$, otherwise the sample is assigned to ${\boldsymbol{\Gamma}}^{\left(+\right)}$. **a** GRNN output based on ${\boldsymbol{K}}_1/{\boldsymbol{k}}_2$ parameter vector aligned with sample grouping described above. Note that all members of ${\boldsymbol{\Gamma}}^{\left(-\right)}$ and all but one of the normal samples are correctly classified with some confusion on the HCC samples with a correct classification rate of 87 %. **b** GRNN output on a random permutation of the ${\boldsymbol{K}}_1/{\boldsymbol{k}}_2$ parameter vector showing poor overall classification performance. Only 1 out of 1000 permutations of the ${\boldsymbol{K}}_1/{\boldsymbol{k}}_2$ parameter vector had a correct classification rate greater than 86 %, which resulted in an empirical *p*-value of 0.001 for the observed classification pattern shown in panel A

The correlation between ${\omega}_{mRNA}^{\left({K}_1/{k}_2\right)}$ and the *K*_1_/*k*_2_ PET parameter suggests the expression phenotypes represented by *Γ*^(−)^ and *Γ*^(+)^ can be distinguished by the *K*_1_/*k*_2_ parameter [42, 59, 60]. To test this hypothesis, we encoded the information content of the *K*_1_/*k*_2_ parameter vector in a Generalized Regression Neural Network (GRNN) implemented in MATLAB (The MathWorks Inc., Natick, MA) after denoising by the Daubechies mother wavelet of order 3 over 5 scales [61–63]. The GRNN model was trained using a ‘spread” parameter set at 0.23235 that defines the level of smoothing of the GRNN output. Training of the GRNN was supervised by a binary target vector, *T* ∈ {0, 1}^50^, where the samples in *Γ*^(+)^ and *Γ*^(−)^ were labeled with a “0” and “1”, respectively. Figure 12a visualizes the output of a GRNN trained on the *K*_1_/*k*_2_ parameter for the 50 samples included in this study. Samples of the expression phenotype *Γ*^(−)^ are highlighted by red squares (ICC), green triangles (CL-HCC) and black asterisks (sarcoma) while the samples in *Γ*^(+)^ (adjacent-normal and HCC) are highlighted as blue circles. The horizontal threshold (magenta line) was used to classify each of the 50 samples by assigning a sample to the *Γ*^(−)^ phenotype if its GRNN value was greater than the threshold and to *Γ*^(+)^ otherwise. Here, we see the GRNN trained on the denoised *K*_1_/*k*_2_ vector correctly classified all the samples in *Γ*^(−)^ and 71 % of the samples in *Γ*^(+)^ for an average correct classification rate of 86 %, which is significantly greater than chance. We note that the GRNN output vector was significantly correlated with the target values in *T* (*r* = 0.61267 *p* = 1.987*E* − 06). To assess the robustness of this result, the *K*_1_/*k*_2_ parameter vector was randomly permuted 1000 times and a GRNN was trained on each permutation using the target vector *T* and spread parameter equal to 0.23235*.* Figure 12b shows that it is difficult to separate *Γ*^(−)^ and *Γ*^(+)^ using any threshold on the output of a GRNN trained on a random permutation of the *K*_1_/*k*_2_ parameter vector, which is reflected in the low correlation of the GRNN output with the target vector *T*$\left(r=0.27615,\;p=0.05223\right)$. Out of 1000 permutations only one had correlation greater than *r* = 0.61, which resulted in an empirical *p*-value of ${p}_{K_1/{k}_2}=1/1000=0.001$. Hence, the observed separation of *Γ*^(−)^ and *Γ*^(+)^ shown in Fig. 12a was probably not a random event.

These preliminary results suggest that the non-invasive monitoring of specific biological processes over time in liver tumors using PET imaging is possible. Note the *K*_1_/*k*_2_ kinetic parameter is just one of many quantitative features that can be extracted from PET images for the supervised analysis of genomic data sets. Relating predictive signatures extracted from molecular images to global patterns of genomic, transcriptomic, epigenomic and metabolomic variation using algorithms such as JAMMIT can be referred to as “imaging genomics” [42, 64]. The central hypothesis of imaging genomics is that image features that capture variation over space and time reflect underlying genetic programs of biological and clinical relevance.

## Discussion and conclusions

We have demonstrated that if the support of a dominant SOI of a big MMDS is supported by a small percentage of all measured variables, then *ℓ*_1_ regularization provides an efficient and powerful way to identify this sparse signature. We encoded this approach in the Joint Analysis of Many Matrices by ITeration (JAMMIT) algorithm that estimates a sparse signal model using an implementation of the LASSO that regularizes the best rank-1 matrix approximation of the super-matrix that vertically “stacks” the individual data matrices of a MMDS based on the *ℓ*_1_ norm. By unstacking the super-signature derived by JAMMIT we obtain type-specific signatures that characterize clinically important attributes of the samples in terms of variables of one or more data types. JAMMIT compared favorably with other joint analysis algorithms in the detection of multiple SOI embedded in simulated MMDS over a wide range of SNR scenarios. Application of JAMMIT to ovarian cancer from TCGA resulted in novel, low-dimensional signatures that linked overall survival to host immune response and macrophage polarization in the tumor microenvironment. We also demonstrated that multi-modal signatures composed of mRNA and methylation variables can result in predictive models of overall survival that outperform models based on uni-modal signatures composed of only mRNA or DNA methylation variables alone. Finally, JAMMIT analysis of whole-genome mRNA and PET imaging data for liver cancer revealed a novel sub-type of HCC with an expression signature similar to that of ICC, a tumor sub-type with a much poorer clinical outcome. Pathway analysis indicated that this expression signature was associated with a pervasive down-regulation of genes and pathways that regulated membrane transport of lipids, suggesting that any difference in clinical outcome between these two tumor subtypes may be due in part to membrane transport dysregulation. This particular application of JAMMIT to liver cancer also demonstrates how the analysis of a single big data matrix can be supervised by an arbitrary univariate function using *ℓ*_1_ regularization.

In developing the JAMMIT algorithm we encountered a number of technical issues related to the joint analysis of multiple data types that will require further study. For example, we have shown that *ℓ*_1_ regularization of the super-matrix that vertically stacks multiple, big data matrices of a MMDS for ovarian cancer resulted in low-dimensional, multi-modal signatures that were biologically coherent and predictive of clinical outcomes. For this analysis, each data matrix was appropriately pre-processed as a function of data type, and the resulting super-matrix was scaled by its Frobenius norm. The sensitivity of JAMMIT-derived signatures to this front-end pre-processing procedure is an open question that will be answered more definitively in future studies. Another issue pertains to systematic variation in the data that we assume is unique to a given data type. Since JAMMIT models a dominant source of common variation that is shared across multiple data types, we expect the FDR profiles of each data type to rapidly decrease in unison as a function of increasing *ℓ*_1_ penalty for such a signal.. In this case, it is unlikely that the resulting signal model represents systematic variation that is by definition unique to a single data type. Alternatively, if only a single data type shows a rapidly decreasing FDR profile, then it is likely that JAMMIT is modeling a source of systematic variation that is unique to that data type. Subsequent downstream processing of the resulting type-specific signatures using pathway and ontological analysis should be able to resolve some of the ambiguity regarding the biological and/or clinical relevance of such signatures. This feature of JAMMIT to discriminate between systematic and biologically relevant sources of variation based on FDR decay will be characterized more fully in future investigations. Finally, the use of FDR to select an appropriate *ℓ*_1_ penalty that balances statistical significance and signature size provides researchers with considerable flexibility in model selection, but it comes with a high computational cost associated with permutation testing. Future studies should consider alternative methods of selecting an “optimal” *ℓ*_1_ penalty that takes into account user preferences for model parsimony, sensitivity, and specificity without the need for resampling.

This study illustrates the importance of taking a sequential approach to data reduction that incorporates biological knowledge in a computational model at the appropriate time to enable robust predictions in larger populations. For example, the use of prior biological knowledge encoded in IPA to “decompose” a given JAMMIT signature into smaller sub-signatures based on significant upstream regulators was shown to result in low-dimensional signatures of clinical significance that facilitated downstream biological interpretation and validation. In general, the reduction of big, multi-modal data sets to low-dimensional signatures that accurately model the clinical trajectory of cancer and other complex diseases can be realized by incorporating biological knowledge at appropriate points in the modeling process where algorithms such as JAMMIT represent just the first step of the data reduction process.

## Additional files

Additional file 1: Estimating FDR profiles on a grid of *ℓ*_1_ penalties. (DOCX 59 kb) Additional file 2: Generation of simulated MMDS. (DOCX 86 kb) Additional file 3: Eigen-survival modeling of JAMMIT signatures. (DOCX 42 kb) Additional file 4: FDR profile of a JAMMIT analysis of multi-modal data for ovarian cancer from TCGA. This table summarizes the relationship between *ℓ*_1_ penalties and FDR that is estimated based on 100 permutations of the super-matrix of a MMDS for ovarian cancer that integrates whole-genome mRNA, miRNA and DNA methylation data obtained from 291 patients with stage3 disease. Note the FDR profiles for each data type (columns 4, 6, and 8) are decreasing towards smaller values indicating that all 3 data types contribute to some degree to a “sparse” linear model of the SOI, with mRNA contributing the most in terms of FDR. In particular, row 19 (in red) is highlighted since it corresponds to a FDR for mRNA of 0.0034619 that is a local minimum of column 4. This FDR value is associated with an *ℓ*_1_ penalty of 0.002875 that results in a mRNA signature composed of 643 genes (FDR=0.0034619), a miRNA signature of 368 miRNAs (FDR=0.19912), a methylation signature of 450 methylation loci (FDR=0.03038), and a multi-modal signature composed of a 1461 variables (FDR=0.067647). (DOCX 20 kb) Additional file 5: FDR profile for analysis of whole-genome gene expression data supervised by the *K*_1_/*k*_2_ PET parameter. Note the *K*_1_/*k*_2_ PET parameter (column 5) is selected for inclusion in the sparse linear model of the SOI for most *ℓ*_1_ penalties with FDR values of zero. Moreover, the FDR profile for genes (column 4) is rapidly decreasing indicating a strong signature for gene expression. These results taken together suggest that the *K*_1_/*k*_2_ parameter is associated with gene expression via the sparse linear model for the SOI. In particular, row 12 (highlighted in red) corresponds to a FDR for mRNA of 0.00054949 that is a local minimum of column 4. This FDR value is associated with a *ℓ*_1_ penalty of 0.0089429 that results in a mRNA signature composed of 652 genes. (DOCX 19 kb) Additional file 6: FDR profile for analysis of whole-genome expression data supervised by the *K*_1_ PET parameter. This FDR profile indicates a lack of correlation between global gene expression and the *K*_1_ PET kinetic parameter. Note that the *K*_1_ PET parameter (column 5) is NOT selected for inclusion in the model of the SOI for all but the first *ℓ*_1_ penalty value (see row 1) with FDR values of 1.0. This result is in sharp contrast to the FDR profile for gene expression (column 4) where the FDR values rapidly decrease to small values. This result suggests that although there is a strong signal in the mRNA data matrix that contributes to the common SOI, this signal is not correlated with the *K*_1_ PET parameter. (DOCX 19 kb)

## Abbreviations

2TC model: 2-Tissue Compartmental model
AUROC: Area Under the ROC
BEST: Bet on Sparsity Principle
CCA: Canonical Correlation Analysis
ESM: Eigen-Survival Model
FDR: False Discovery Rate
GVSD: Generalized Singular Value Decomposition
HCC: HepatoCellular Carcinoma
ICC: Intra-hepatic CholangioCarcinoma
IPA: Ingenuity Pathway Analysis
JAMMIT: Joint Analysis of Many Matrices by ITeration
JIVE: Joint and Individual Variation Explained
LASSO: Least Absolute Shrinkage and Selection Operator
LOOCV: Leave-One-Out Cross-Validation)
MMDS: Muti-Modal Data Set
MMSIG: Multi-Modal Signature
mRNA: messenger RNA
PET/CT: Positron Emission Tomography/Computed Tomography)
PLS: Partial Least Squares
ROC: Receiver Operater Characteristic
SNR: Signal-to-Noise Ratio
SOI: Signal of Interest
TCGA: The Cancer Genome Atlas
