# Supplementary material for: Joint analysis of multiple high-dimensional data types using sparse matrix approximations of rank-1 with applications to ovarian and liver cancer
Source: BioData Min. 2016 Jul 29;9:24. doi: 10.1186/s13040-016-0103-7 (PMC4966782; doi:10.1186/s13040-016-0103-7)
Supplement: Additional file 1: — Estimating FDR profiles on a grid of ℓ 1 penalties. (DOCX 59 kb) [file 13040_2016_103_MOESM1_ESM.docx]

**Additional file 1. Estimating FDR profiles on a grid of penalties.**

The following workflow summarizes the generation of a sequence of FDR values based on a monotone increasing sequence of $\lambda$’s:

1. Let $\Lambda=\left\{0=\lambda_{1}<\lambda_{2}<\ldots<\lambda_{l}<\ldots<\lambda_{\sup}<\infty\right\}$ be a montonically increasing sequence of $\mathcal{l}_{1}$ penalties such that for all $\lambda\in\Lambda$ we have $\left| \zeta\left( \lambda\right) \right|=0$ only if $\lambda>\lambda_{sup}$ where $\zeta\left( \lambda\right)$ is the JAMMIT-derived signature for the $\mathcal{l}_{1}$ penalty $\lambda$.
2. Generate a collection of permuted matrices $\Delta=\left\{D^{\left(b\right)}\right\}_{b=0}^{B}$ based on the original super-matrix $D$ where $D^{\left(0\right)}\equiv D$and each $D^{\left(b\right)}$ is obtained by randomly permuting each row of$D$ for $b=1,2,\ldots,B$.
3. For a given $\lambda\in\Lambda$
   1. Apply JAMMIT to matrix $D^{\left(b\right)}\in\Delta$ and compute $s^{\left(b\right)}\left(\lambda\right)=\left|\zeta^{\left(b\right)}\left(\lambda\right)\right|$ for $b=0,1,2,\ldots,B$ where $\zeta^{\left(0\right)}\left(\lambda\right)$ is the JAMMIT signature for $D$ and $\zeta^{\left(b\right)}\left(\lambda\right)$ is the JAMMIT signature for $D_{k}^{\left(b\right)}$ for $k=1,2,\cdots,K$.
   2. Compute $s^{\left(med\right)}\left(\lambda\right)=median\left(\left\{s^{\left(b\right)}\left(\lambda\right)\right\}_{b=1}^{B}\right)$
   3. Compute $\Theta\left(\lambda\right)=\pi_{0}\left(s^{\left(med\right)}\left(\lambda\right)/s^{\left(0\right)}\left(\lambda\right)\right)$ where $\pi_{0}$ is an estimate of the true proportion of non-zero loading coefficients of $u$ (usually set at $\pi_{0}=0.05$).
4. Repeat step (2) for each $\lambda\in\Lambda$ to define a joint FDR sequence $\Theta:\Lambda\mapsto[0,1]$.
5. Define FDR sequences $\Theta_{k}:\Lambda\to[0,1]$ for each $D_{k}$ where $\Theta_{k}\left(\lambda\right)$ is equal to $\Theta\left(\lambda\right)$ restricted to the data matrix $D_{k}$.
