# Supplementary material for: Joint analysis of multiple high-dimensional data types using sparse matrix approximations of rank-1 with applications to ovarian and liver cancer
Source: BioData Min. 2016 Jul 29;9:24. doi: 10.1186/s13040-016-0103-7 (PMC4966782; doi:10.1186/s13040-016-0103-7)
Supplement: Additional file 2: — Generation of simulated MMDS. (DOCX 86 kb) [file 13040_2016_103_MOESM2_ESM.docx]

**Additional file 2. Generation of simulated MMDS**

Each $D_{k}$ was additively modeled by $D_{k}=\Sigma_{k}+N_{k}$ for $k=1,2$ where $\Sigma_{k}$ and $N_{k}$ are $p_{k}\times50$ signal-only and noise-only data matrices, respectively, that were simulated as follows:

1. Generate the $p_{k}\times50$ matrix, $\Sigma_{k}$, composed of zeros.
2. Let $I_{k}\left(step\right)$ and $I_{k}\left(rand\right)$ be indicator functions that randomly select 2 subsets of row-indices from $\Sigma_{k}$, denoted by $Supp_{k}\left(step\right)$and $Supp_{k}\left(rand\right)$, such that: a)$Supp_{k}\left(step\right)\cap Supp_{k}\left(rand\right)=\emptyset$; and b) $\left|Supp_{k}\left(step\right)\right|$+$\left|Supp_{k}\left(rand\right)\right|$ << $p_{i}$
3. For each $i\in Supp_{k}\left(step\right)$ replace the *i*th row of $\Sigma_{k}$ with defined by

$$step_{i}=\left\{\begin{matrix} -\alpha_{i}\text{, }j=1,2,\ldots,25 \\ +\alpha_{i}\text{, }j=26,\ldots,50 \end{matrix}\right.$$

where $\alpha_{i}\in\Lambda_{step}$ for some predetermined interval of positive real numbers $\Lambda_{step}$. The collection of rows of $\Sigma_{k}$ that are replaced by $step_{i}$ define a multiplexed, step-signal,, that is supported by the rows in $Supp_{k}\left(step\right)$ over the 50 columns of $\Sigma_{k}$. Note the variance of the rows of the multiplexed signal, $mstep_{i}$, varies with the amplitude of $step_{i}$.

1. For each $i\in Supp_{k}\left(rand\right)$ replace the *i*th row of $\Sigma_{k}$ with the random vector with components sampled from a zero-mean, Gaussian distribution with variance $\sigma_{i}^{2}$. The collection of rows of $\Sigma_{k}$ that contain $rand_{i}$ define a multiplexed, random-signal,, that is supported by the rows in $Supp_{i}\left(rand\right)$over the 50 columns of $\Sigma_{k}$. Note the variance of the rows of the multiplexed signal, $mrand_{i}$, varies with $\sigma_{i}^{2}$.
2. For $k=1,2$, define $N_{k}$ as the $p_{k}\times50$ random matrix with entries from a zero-mean Gaussian distribution of variance $\sigma_{k}$ .
3. For $k=1,2$, define $D_{k}=\Sigma_{k}+N_{k}$

Steps 1-6 were repeated 1000 times where each repetition resulted in a MMDS, $D^{\left(\eta\right)}=\left\{D_{k}^{\left(\eta\right)}\right\}_{k=1}^{2}=\left\{\left(\Sigma_{k}^{\left(\eta\right)}+N_{k}^{\left(\eta\right)}\right)\right\}_{k=1}^{2}$, for $\eta=1,2,\ldots,1000$.
