# Supplementary material for: Joint analysis of multiple high-dimensional data types using sparse matrix approximations of rank-1 with applications to ovarian and liver cancer
Source: BioData Min. 2016 Jul 29;9:24. doi: 10.1186/s13040-016-0103-7 (PMC4966782; doi:10.1186/s13040-016-0103-7)
Supplement: Additional file 3: — Eigen-survival modeling of JAMMIT signatures. (DOCX 42 kb) [file 13040_2016_103_MOESM3_ESM.docx]

**Additional file 3 - Eigen-survival modeling of JAMMIT signatures**

Let $\zeta\left(D\right)$ be a JAMMIT-derived signature in the $p\times n$ data matrix $D$ that was decomposed using the SVD to obtain the outer-product representation

$\zeta\left(D\right)=\sum_{r=1}^{R} s_{r}\left(u_{r}v_{r}^{T}\right)$ (1)

where: i) , , and are the *r*th singular value, left –singular vector, and right-singular value, respectively, for $r=1,2,...,R$; and ii) $R$ is the rank of $\zeta\left(D\right)$. Each $v_{r}$, in (1) was tested for association with the survival data in $\Omega\left(D\right)$ using Kaplan-Meier (KM) analysis with log-rank testing, and Cox regression modeling with age as a covariate. To accomplish this, we interpreted the $n$ components of $v_{r}$ as “prognostic scores” for each patient and sorted the patients by this score to identify those that fell in the top and bottom quartiles of scores. A given $v_{r}$ was called significant if and only if differences in survival between patients in top and bottom quartiles based on $v_{r}$ are significant in both the KM and Cox regression models with a p-value of 0.05 or less. Given that at least one such $v_{r}$ exists, we define: 1) $B=\{r | v_{r} \text{is significant in both the KM and Cox models}\}$; 2) $U=\left\{ u_{r} \right| r\in B\}$; and 3) $V=\left\{ v_{r} \right| r\in B\}$.

Then the eigen-survival model, , based on $\zeta\left(D\right)$ is defined by the linear combination of the vectors in $V$:

$\Phi=\sum_{r\in B} sign\left( r \right)s_{r}v_{r}$ (10)

where $sign\left( r \right)= \pm1$ was chosen for each $r\in B$ so that the association of (10) with overall survival is maximized in terms of p-values of in KM and Cox regression models. Note that $\Phi$ can also be expressed in terms of singular vectors in $U$ by

$\Phi^{T}= \sum_{r\in B} sign\left( r \right) u_{r}^{T}\zeta(D)$ (11A)

or equivalently

$\Phi= \sum_{r\in B} sign\left( r \right) \zeta\left( D \right)^{T}u_{r}$. (11B)

In other words, the $j$th entry of $\Phi$, i.e., the prognostic score for the $j$th patient, is the dot product of the $j$th column of $\zeta\left( D \right)$, which consists of the measurements of the variables in the signature for that patient, with the test vector $\Gamma= \sum_{r\in B} sign\left( r \right) u_{r}$.

To compute prognostic scores for a set of new patients not included in the original samples, let $\zeta(n')$ be an $s\times n'$ matrix with columns that represent realizations of $\zeta$ for $n'$ patients that were unseen during discovery of $\zeta$. Then following (11B) we form

$\Phi'= \sum_{r\in B} sign\left( r \right) \zeta\left( n^{'} \right)^{T}u_{r}$ (12)

which transforms the columns of $\zeta(n^{'})$ into prognostic scores for these patients based on the eigen-survival model defined by (12B). If KM and Cox regression analysis indicates that $\Phi'$ is significantly associated with overall survival, then we conclude that the eigen-survival model defined by (11B) can be generalized to a larger population beyond the original $n$ patients that were used to discover $\zeta$.
