# Supplementary material for: Joint analysis of multiple high-dimensional data types using sparse matrix approximations of rank-1 with applications to ovarian and liver cancer
Source: BioData Min. 2016 Jul 29;9:24. doi: 10.1186/s13040-016-0103-7 (PMC4966782; doi:10.1186/s13040-016-0103-7)
Supplement: Additional file 4: — FDR profile of a JAMMIT analysis of multi-modal data for ovarian cancer from TCGA. This table summarizes the relationship between ℓ 1 penalties and FDR that is estimated based on 100 permutations of the super-matrix of a MMDS for ovarian cancer that integrates whole-genome mRNA, miRNA and DNA methylation data obtained from 291 patients with stage3 disease. Note the FDR profiles for each data type (columns 4, 6, and 8) are decreasing towards smaller values indicating that all 3 data types contribute to some degree to a “sparse” linear model of the SOI, with mRNA contributing the most in terms of FDR. In particular, row 19 (in red) is highlighted since it corresponds to a FDR for mRNA of 0.0034619 that is a local minimum of column 4. This FDR value is associated with an ℓ 1 penalty of 0.002875 that results in a mRNA signature composed of 643 genes (FDR=0.0034619), a miRNA signature of 368 miRNAs (FDR=0.19912), a methylation signature of 450 methylation loci (FDR=0.03038), and a multi-modal signature composed of a 1461 variables (FDR=0.067647). (DOCX 20 kb) [file 13040_2016_103_MOESM4_ESM.docx]

Additional file 4. FDR profile of mRNA signature for ovarian cancer

| **(1)**  **Row number** | **(2)**  **l1 penalty**  $\boldsymbol{\lambda}$ | **(3)**  **# of selected mRNAs** | **(4)**  **FDR (mRNA)** | **(5)**  **# of selected miRNAs** | **(6)**  **FDR (miRNA)** | **(7)**  **# of selected methylation loci** | **(8)**  **FDR (meth))** | **(9)**  **Total # of selected variables** | **(10)**  **FDR (total)** |
| --- | --- | --- | --- | --- | --- | --- | --- | --- | --- |
| 1 | 0.001 | 6081 | 0.073801 | 497 | 0.46576 | 3000 | 0.2213 | 9578 | 0.1432 |
| 2 | 0.0011042 | 5408 | 0.058633 | 485 | 0.45275 | 2701 | 0.20162 | 8594 | 0.1289 |
| 3 | 0.0012083 | 4806 | 0.047401 | 471 | 0.42646 | 2450 | 0.17776 | 7727 | 0.11503 |
| 4 | 0.0013125 | 4249 | 0.039479 | 461 | 0.4118 | 2213 | 0.16372 | 6923 | 0.1074 |
| 5 | 0.0014167 | 3766 | 0.032858 | 455 | 0.38716 | 2036 | 0.14376 | 6257 | 0.098243 |
| 6 | 0.0015208 | 3372 | 0.026972 | 451 | 0.35987 | 1841 | 0.12738 | 5664 | 0.089743 |
| 7 | 0.001625 | 3000 | 0.02226 | 442 | 0.34352 | 1662 | 0.11666 | 5104 | 0.084613 |
| 8 | 0.0017292 | 2681 | 0.018874 | 439 | 0.32977 | 1516 | 0.10182 | 4636 | 0.079444 |
| 9 | 0.0018333 | 2387 | 0.016503 | 428 | 0.30996 | 1385 | 0.09317 | 4200 | 0.075755 |
| 10 | 0.0019375 | 2124 | 0.014017 | 416 | 0.29888 | 1256 | 0.08454 | 3796 | 0.072804 |
| 11 | 0.0020417 | 1868 | 0.012385 | 412 | 0.27995 | 1119 | 0.07504 | 3399 | 0.069847 |
| 12 | 0.0021458 | 1626 | 0.0099987 | 405 | 0.27814 | 1015 | 0.06824 | 3046 | 0.069875 |
| 13 | 0.00225 | 1451 | 0.0082733 | 398 | 0.26131 | 921 | 0.06123 | 2770 | 0.067138 |
| 14 | 0.0023542 | 1289 | 0.0073703 | 391 | 0.2531 | 830 | 0.05229 | 2510 | 0.065662 |
| 15 | 0.0024583 | 1131 | 0.0068184 | 388 | 0.23449 | 734 | 0.05003 | 2253 | 0.065395 |
| 16 | 0.0025625 | 1004 | 0.0055825 | 385 | 0.22469 | 654 | 0.04387 | 2043 | 0.064688 |
| 17 | 0.0026667 | 877 | 0.0050764 | 380 | 0.21641 | 576 | 0.03767 | 1833 | 0.06503 |
| 18 | 0.0027708 | 755 | 0.0047384 | 375 | 0.20567 | 511 | 0.03158 | 1641 | 0.065201 |
| ***19*** | ***0.002875*** | ***643*** | ***0.0034619*** | ***368*** | ***0.19912*** | ***450*** | ***0.03038*** | ***1461*** | ***0.067647*** |
| 20 | 0.0029792 | 552 | 0.0036006 | 358 | 0.18865 | 382 | 0.03027 | 1292 | 0.069655 |
| 21 | 0.0030833 | 468 | 0.002718 | 355 | 0.17477 | 339 | 0.02638 | 1162 | 0.069229 |
| 22 | 0.0031875 | 394 | 0.0028249 | 343 | 0.17364 | 294 | 0.02595 | 1031 | 0.073874 |
| 23 | 0.0032917 | 342 | 0.0022084 | 336 | 0.17122 | 257 | 0.02211 | 935 | 0.076543 |
| 24 | 0.0033958 | 295 | 0.0018865 | 332 | 0.1599 | 222 | 0.02041 | 849 | 0.076782 |
| 25 | 0.0035 | 260 | 0.0022933 | 329 | 0.14848 | 186 | 0.01838 | 775 | 0.076545 |

This table summarizes the relationship between a monotonically increasing sequence of $\mathcal{l}_{1}$ penalties and FDR that is estimated based on 100 permutations of the super-matrix of the ovarian MMDS. Row 19 is highlighted since it corresponds to a FDR of 0.0034619 that is a local minimum for an mRNA signature composed of 643 genes and a $\mathcal{l}_{1}$ penalty of 0.002875. This $\mathcal{l}_{1}$ penalty also resulted in a miRNA signature of 368 microRNAs with an FDR of 0.19912, a methylation signature of 450 methylation loci with an FDR of 0.03038, and a MMSIG composed of 1461 mRNA, miRNA and methylation variables with a total FDR of 0.067647.
