# Supplementary material for: Joint analysis of multiple high-dimensional data types using sparse matrix approximations of rank-1 with applications to ovarian and liver cancer
Source: BioData Min. 2016 Jul 29;9:24. doi: 10.1186/s13040-016-0103-7 (PMC4966782; doi:10.1186/s13040-016-0103-7)
Supplement: Additional file 5: — FDR profile for analysis of whole-genome gene expression data supervised by the K 1/k 2 PET parameter. Note the K 1/k 2 PET parameter (column 5) is selected for inclusion in the sparse linear model of the SOI for most ℓ 1 penalties with FDR values of zero. Moreover, the FDR profile for genes (column 4) is rapidly decreasing indicating a strong signature for gene expression. These results taken together suggest that the K 1/k 2 parameter is associated with gene expression via the sparse linear model for the SOI. In particular, row 12 (highlighted in red) corresponds to a FDR for mRNA of 0.00054949 that is a local minimum of column 4. This FDR value is associated with a ℓ 1 penalty of 0.0089429 that results in a mRNA signature composed of 652 genes. (DOCX 19 kb) [file 13040_2016_103_MOESM5_ESM.docx]

Additional file 5. FDR profile for ${\boldsymbol{K}_{\boldsymbol{1}}}/{\boldsymbol{k}_{\boldsymbol{2}}}$ signature for liver cancer

| **(1)**  **Row Number** | **(2)**  **l1 penalty** $\boldsymbol{\lambda}$ | **(3)**  **# of selected genes** | **(4)**  **FDR (genes)** | **(5)**  **# of selected PET parms** | **(6)**  **FDR (PET)** | **(7)**  **# of selected variables** | **(8)**  **FDR (total)** |
| --- | --- | --- | --- | --- | --- | --- | --- |
| 1 | 1.00E-05 | 20613 | 0.98581 | 1 | 0 | 20614 | 0.98577 |
| 2 | 0.00082208 | 12393 | 0.364 | 1 | 0 | 12394 | 0.36403 |
| 3 | 0.0016342 | 8270 | 0.16171 | 1 | 0 | 8271 | 0.1618 |
| 4 | 0.0024463 | 5682 | 0.085654 | 1 | 0 | 5683 | 0.085805 |
| 5 | 0.0032583 | 4009 | 0.047712 | 1 | 0 | 4010 | 0.047936 |
| 6 | 0.0040704 | 2943 | 0.026501 | 1 | 0 | 2944 | 0.026829 |
| 7 | 0.0048825 | 2215 | 0.014121 | 1 | 0 | 2216 | 0.01455 |
| 8 | 0.0056946 | 1696 | 0.0074933 | 1 | 0 | 1697 | 0.0080397 |
| 9 | 0.0065067 | 1322 | 0.0041554 | 1 | 0 | 1323 | 0.0048742 |
| 10 | 0.0073187 | 1052 | 0.0020717 | 1 | 0 | 1053 | 0.0029486 |
| 11 | 0.0081308 | 822 | 0.0010654 | 1 | 0 | 823 | 0.0022007 |
| ***12*** | ***0.0089429*** | ***652*** | ***0.00054949*** | ***1*** | ***0*** | ***653*** | ***0.0018897*** |
| 13 | 0.009755 | 534 | 0.00026091 | 1 | 0 | 535 | 0.0019159 |
| 14 | 0.010567 | 442 | 0.00038277 | 1 | 0 | 443 | 0.002179 |
| 15 | 0.011379 | 373 | 0.00021345 | 1 | 0 | 374 | 0.0024213 |
| 16 | 0.012191 | 327 | 0.00012174 | 1 | 0 | 328 | 0.0024575 |
| 17 | 0.013003 | 279 | 3.57E-05 | 1 | 0 | 280 | 0.00263 |
| 18 | 0.013815 | 226 | 0 | 1 | 0 | 227 | 0.0032441 |
| 19 | 0.014628 | 198 | 0 | 1 | 0 | 199 | 0.0034505 |
| 20 | 0.01544 | 165 | 0 | 1 | 0 | 166 | 0.0037168 |
| 21 | 0.016252 | 133 | 0 | 1 | 0 | 134 | 0.0046787 |
| 22 | 0.017064 | 104 | 0 | 1 | 0 | 105 | 0.0058761 |
| 23 | 0.017876 | 82 | 0 | 0 | 1 | 82 | 0.0093446 |
| 24 | 0.018688 | 58 | 0 | 0 | 1 | 58 | 0.012182 |
| 25 | 0.0195 | 43 | 0 | 0 | 1 | 43 | 0.017589 |

Note the ${K_{1}}/{k_{2}}$ PET parameter (column 5) is selected for inclusion in the sparse model of the SOI for most $\mathcal{l}_{1}$ penalties with FDR values of zero. Moreover, the FDR profile for genes (column 4) is rapidly decreasing indicating a strong signature for gene expression. These results taken together suggest that the ${K_{1}}/{k_{2}}$ parameter is associated with gene expression via the sparse linear model for the SOI. In particular, row 12 (highlighted in red) corresponds to a FDR for mRNA of 0.00054949 that is a local minimum of column 4. This FDR value is associated with an $\mathcal{l}_{1}$ penalty of 0.0089429 that results in a mRNA signature composed of 652 genes.
