# Supplementary material for: Joint analysis of multiple high-dimensional data types using sparse matrix approximations of rank-1 with applications to ovarian and liver cancer
Source: BioData Min. 2016 Jul 29;9:24. doi: 10.1186/s13040-016-0103-7 (PMC4966782; doi:10.1186/s13040-016-0103-7)
Supplement: Additional file 6: — FDR profile for analysis of whole-genome expression data supervised by the K 1 PET parameter. This FDR profile indicates a lack of correlation between global gene expression and the K 1 PET kinetic parameter. Note that the K 1 PET parameter (column 5) is NOT selected for inclusion in the model of the SOI for all but the first ℓ 1 penalty value (see row 1) with FDR values of 1.0. This result is in sharp contrast to the FDR profile for gene expression (column 4) where the FDR values rapidly decrease to small values. This result suggests that although there is a strong signal in the mRNA data matrix that contributes to the common SOI, this signal is not correlated with the K 1 PET parameter. (DOCX 19 kb) [file 13040_2016_103_MOESM6_ESM.docx]

Additional file 6. FDR profile for the $\boldsymbol{K}_{\boldsymbol{1}}$ signature for liver cancer

| **(1)**  **Row Number** | **(2)**  **l1 penalty** $\boldsymbol{\lambda}$ | **(3)**  **# of Selected Genes** | **(4)**  **FDR (Genes)** | **(5)**  **# of Selected PET Params** | **(6)**  **FDR (PET)** | **(7)**  **# of Selected Variables (Total)** | **(8)**  **FDR (Total)** |
| --- | --- | --- | --- | --- | --- | --- | --- |
| 1 | 1.00E-05 | 20609 | 0.98622 | 1 | 0 | 20610 | 0.98617 |
| 2 | 0.00082208 | 12395 | 0.38683 | 0 | 1 | 12395 | 0.38682 |
| 3 | 0.0016342 | 8279 | 0.16905 | 0 | 1 | 8279 | 0.16904 |
| 4 | 0.0024463 | 5687 | 0.079955 | 0 | 1 | 5687 | 0.079951 |
| 5 | 0.0032583 | 4017 | 0.039391 | 0 | 1 | 4017 | 0.03939 |
| 6 | 0.0040704 | 2946 | 0.020025 | 0 | 1 | 2946 | 0.020024 |
| 7 | 0.0048825 | 2216 | 0.010886 | 0 | 1 | 2216 | 0.010885 |
| 8 | 0.0056946 | 1697 | 0.0062515 | 0 | 1 | 1697 | 0.0062512 |
| 9 | 0.0065067 | 1325 | 0.0038756 | 0 | 1 | 1325 | 0.0038754 |
| 10 | 0.0073187 | 1054 | 0.0025682 | 0 | 1 | 1054 | 0.0025681 |
| 11 | 0.0081308 | 823 | 0.0021887 | 0 | 1 | 823 | 0.0021886 |
| 12 | 0.0089429 | 653 | 0.0016764 | 0 | 1 | 653 | 0.0016764 |
| 13 | 0.009755 | 535 | 0.0015997 | 0 | 1 | 535 | 0.0015997 |
| 14 | 0.010567 | 442 | 0.0012158 | 0 | 1 | 442 | 0.0012158 |
| 15 | 0.011379 | 375 | 0.0010085 | 0 | 1 | 375 | 0.0010084 |
| 16 | 0.012191 | 327 | 0.00051738 | 0 | 1 | 327 | 0.00051735 |
| 17 | 0.013003 | 279 | 0.00049938 | 0 | 1 | 279 | 0.00049935 |
| 18 | 0.013815 | 228 | 0.00021824 | 0 | 1 | 228 | 0.00021823 |
| 19 | 0.014628 | 198 | 5.03E-05 | 0 | 1 | 198 | 5.03E-05 |
| 20 | 0.01544 | 165 | 6.03E-05 | 0 | 1 | 165 | 6.03E-05 |
| 21 | 0.016252 | 133 | 0 | 0 | 1 | 133 | 0 |
| 22 | 0.017064 | 105 | 0 | 0 | 1 | 105 | 0 |
| 23 | 0.017876 | 82 | 0 | 0 | 1 | 82 | 0 |
| 24 | 0.018688 | 58 | 0 | 0 | 1 | 58 | 0 |
| 25 | 0.0195 | 43 | 0 | 0 | 1 | 43 | 0 |

This FDR profile indicates a lack of correlation between global gene expression and the $K_{1}$ PET kinetic parameter. Note that the $K_{1}$ PET parameter (column 5) is NOT selected for inclusion in the model of the SOI for all but the first $\mathcal{l}_{1}$ penalty value (see row 1) with FDR values of 1.0. This result is in sharp contrast to the FDR profile for gene expression (column 4) where the FDR values rapidly decrease to small values. This result suggests that although there is a strong coherent signature for gene expression that contributes to the common SOI, this signal is not correlated with the $K_{1}$ PET parameter.
